# Supplementary material for: Metronomic chemotherapy offsets HIFα induction upon maximum‐tolerated dose in metastatic cancers
Source: EMBO Mol Med. 2020 Jul 20;12(9):e11416. doi: 10.15252/emmm.201911416 (PMC7507002; doi:10.15252/emmm.201911416)
Supplement: Supplementary file 1 — Appendix [file EMMM-12-e11416-s001.pdf]

Appendix

Metronomic chemotherapy offsets HIFα induction upon maximum-tolerated dose in metastatic cancers

Luana Schito, Sergio Rey, Ping Xu, Shan Man, William Cruz-Muñoz and Robert S. Kerbel

Table of Contents

- Appendix Figure S1: LDM chemotherapy offsets HIF-1α levels in primary colon cancer (extended image set), page 2
- Appendix Figure S2: Effect of LDM and/or MTD chemotherapy upon primary orthotopic colon cancer growth, page 3
- Appendix Figure S3: Correlation among HIF-1α, HIF-2α, CA9 and Ki67 proliferative indexes in primary colon cancer, page 4
- Appendix Figure S4: HIFα expression in colon cancer liver metastases (extended image set), page 5
- Appendix Figure S5: Effect of LDM and/or MTD chemotherapy upon HIF-2α in colon cancer metastases to the lungs (extended image set), page 6
- Appendix Figure S6: Lung parenchymal perfusion in metastatic EMT6-CDDP breast cancer, page 7
- Appendix Figure S7: HIF-1α-dependent proliferative index in breast cancer metastatic nodules to the lungs, page 8
- Appendix Table S1 – List of antibodies, page 9

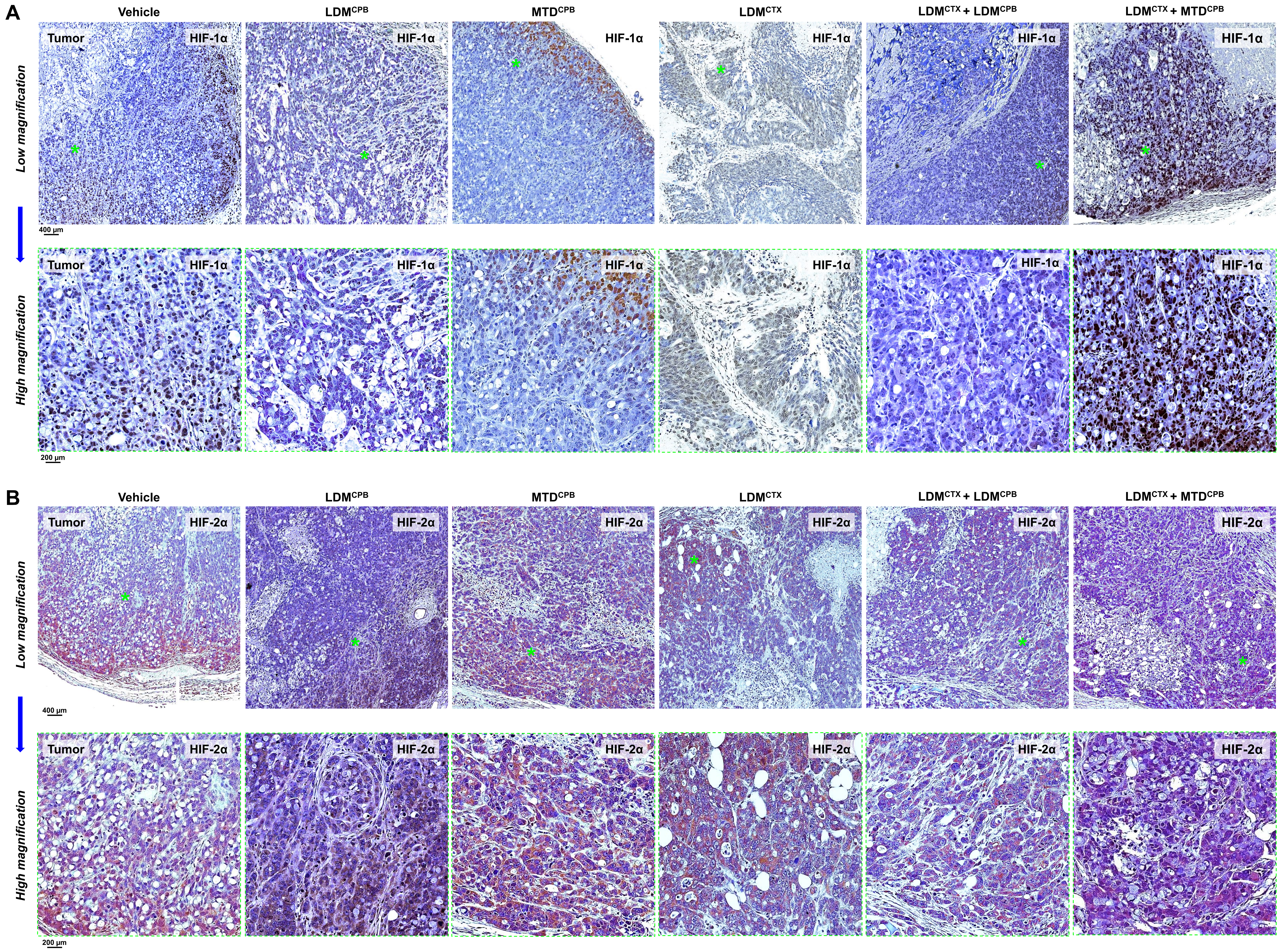

Appendix Figure S1:
LDM chemotherapy offsets HIF-1α levels in primary colon cancer

Extended image set of Figure 1A and 1B, showing low and high magnification images in all experimental groups, including non-statistically significant treatments after automated immunoreactive area quantification.

A: HIF-1α levels in HT29 primary tumors.

Top row: low magnification; *bottom row*: high magnification.

Vehicle, LDM^{CTX} + LDM^{CPB} and LDM^{CTX} + MTD^{CPB} high magnification images in the *bottom row* correspond to Figure 1A (*left*, *center/left* and *center/right*, respectively).

B: HIF-2α levels in HT29 primary tumors.

Top row: low magnification; *bottom row*: high magnification.

High magnification view of the Vehicle group (*left*, *bottom row*) corresponds to the HIF-2α image in Figure 1B.

Data information: CPB, capecitabine; CTX, cyclophosphamide; LDM, low-dose metronomic; MTD, maximum-tolerated dose. High-magnification images are centered on the region marked with a green asterisk (*) within the low magnification rows.

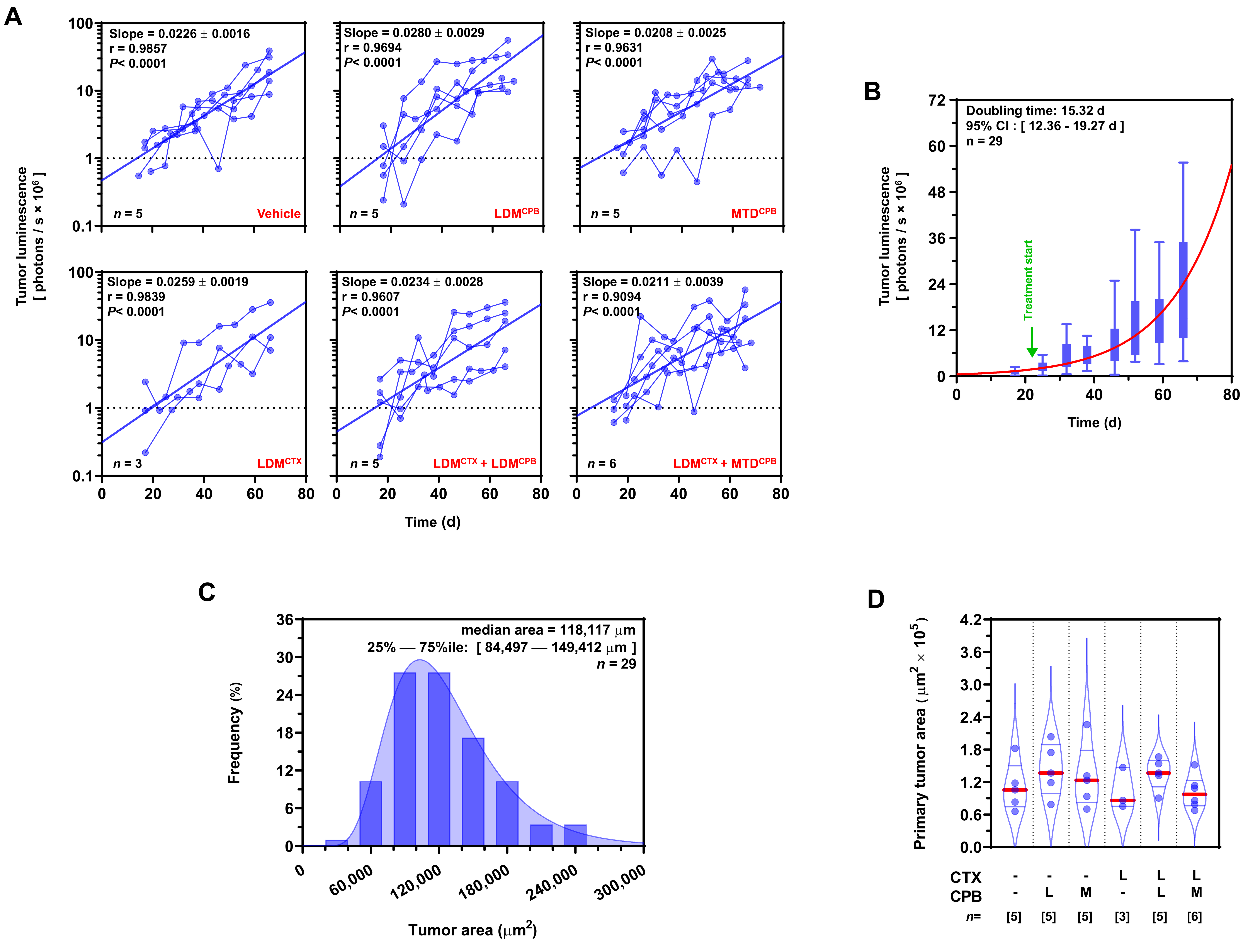

Appendix Figure S2:
Effect of LDM and/or MTD chemotherapy upon primary orthotopic colon cancer growth

A: Luminescent signal time-course from luciferase-expressing HT29 colonic adenocarcinomas.
Each trace corresponds to an individual orthograft wherein photon flux is displayed in a logarithmic scale.
Slope differences were not statistically significant among experimental groups, $F_{(5,220)}$= 0.6791 and P= 0.6397; nonetheless overall slopes were ≠ 0 [pooled: 0.02359; $F_{(1,230)}$ = 264.8 and P< 0.0001 by F-tests].

B: Calculation of HT29 colon adenocarcinoma luminescence doubling time.
Boxes indicate interquartile ranges whilst error bars (i.e., 'whiskers') show *minima* and *maxima*.

C: Cross-sectional area distribution in HT29 primary colonic adenocarcinomas.
Quantification of tumoral area.

D: Effect of LDM and/or MTD chemotherapy upon primary tumor cross-sectional area.
$F_{(5,23)}$ = 0.8226 and P= 0.5464 by one-way Brown-Forsythe ANOVA.

Data information: Violin plots present 50th (red line), 25th and 75th percentiles (blue lines); brackets: n, number of tumors. CPB, capecitabine; CTX, cyclophosphamide; L or LDM, low-dose metronomic; M or MTD, maximum-tolerated dose; r, correlation coefficient.

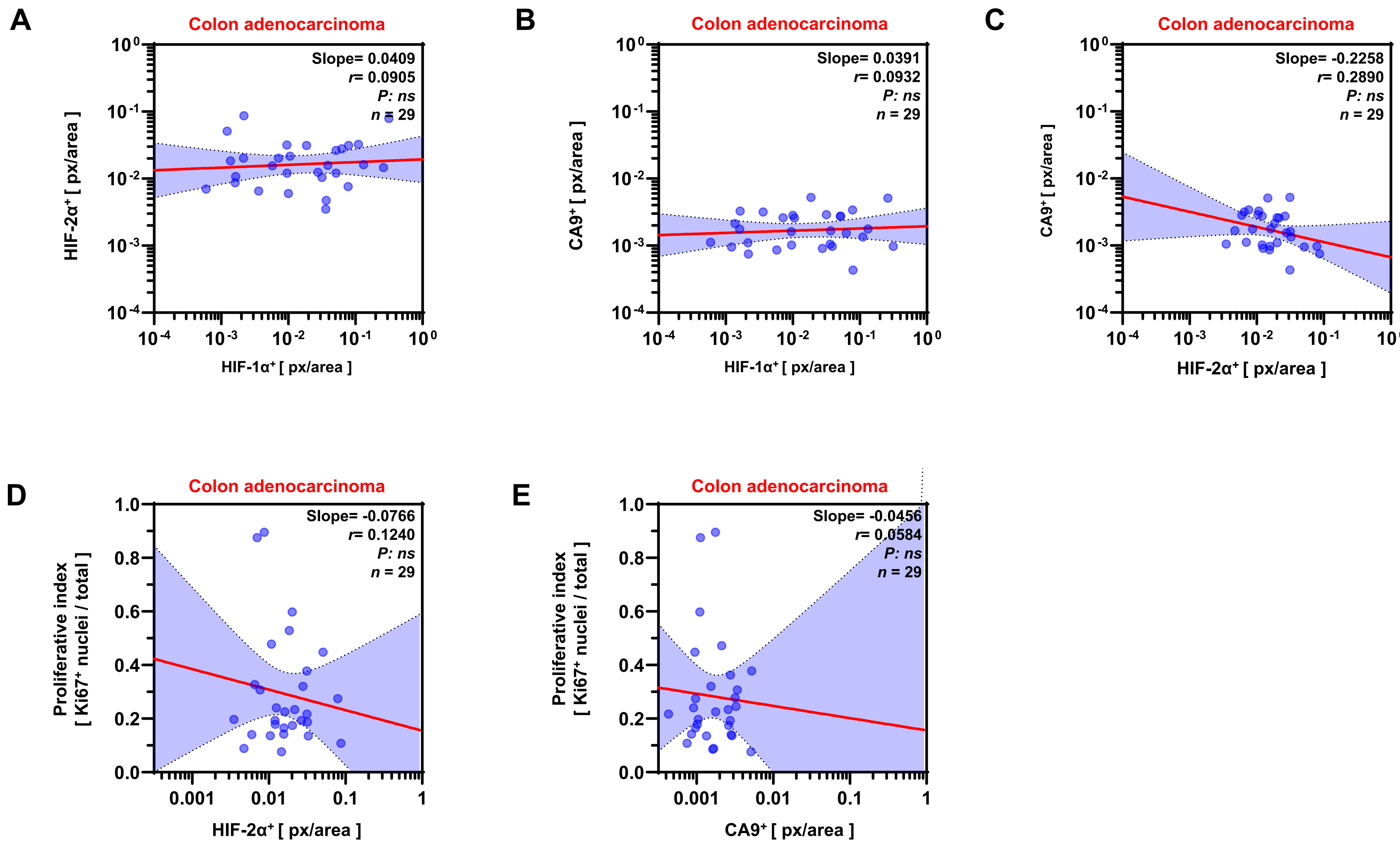

Appendix Figure S3:
Correlation among HIF-1α, HIF-2α, CA9 and Ki67 proliferative indexes in primary colon cancer

A: HIF-1α *vs* HIF-2α $F_{(1,27)}$= 0.2229, *P*= 0.6406 (not significant); slope ≠ 0 by *F*-test.

B: HIF-1α *vs* CA9. $F_{(1,27)}$= 0.2368, *P*= 0.6305 (not significant); slope ≠ 0 by *F*-test.

C: HIF-2α *vs* CA9. $F_{(1,27)}$= 2.460, *P*=0.1284 (not significant); slope ≠ 0 by *F*-test.

D: HIF-2α *vs* proliferative index. $F_{(1,27)}$= 0.4215, *P*=0.5217 (not significant); slope ≠ 0 by *F*-test.

E: CA9 *vs* proliferative index. $F_{(1,27)}$= 0.0925, *P*=0.7634 (not significant); slope ≠ 0 by *F*-test.

Data information: Pearson regression line (red) and 95% CI (shaded blue area) are shown. HIF-1α, HIF-2α and CA9 immunoreactivities are expressed as fractions of tumoral area. Proliferative indexes were calculated by an automated machine-learning algorithm counting Ki67^{+} nuclei fractions; *n*, number of tumors; *ns*, not significant; *r*, correlation coefficient.

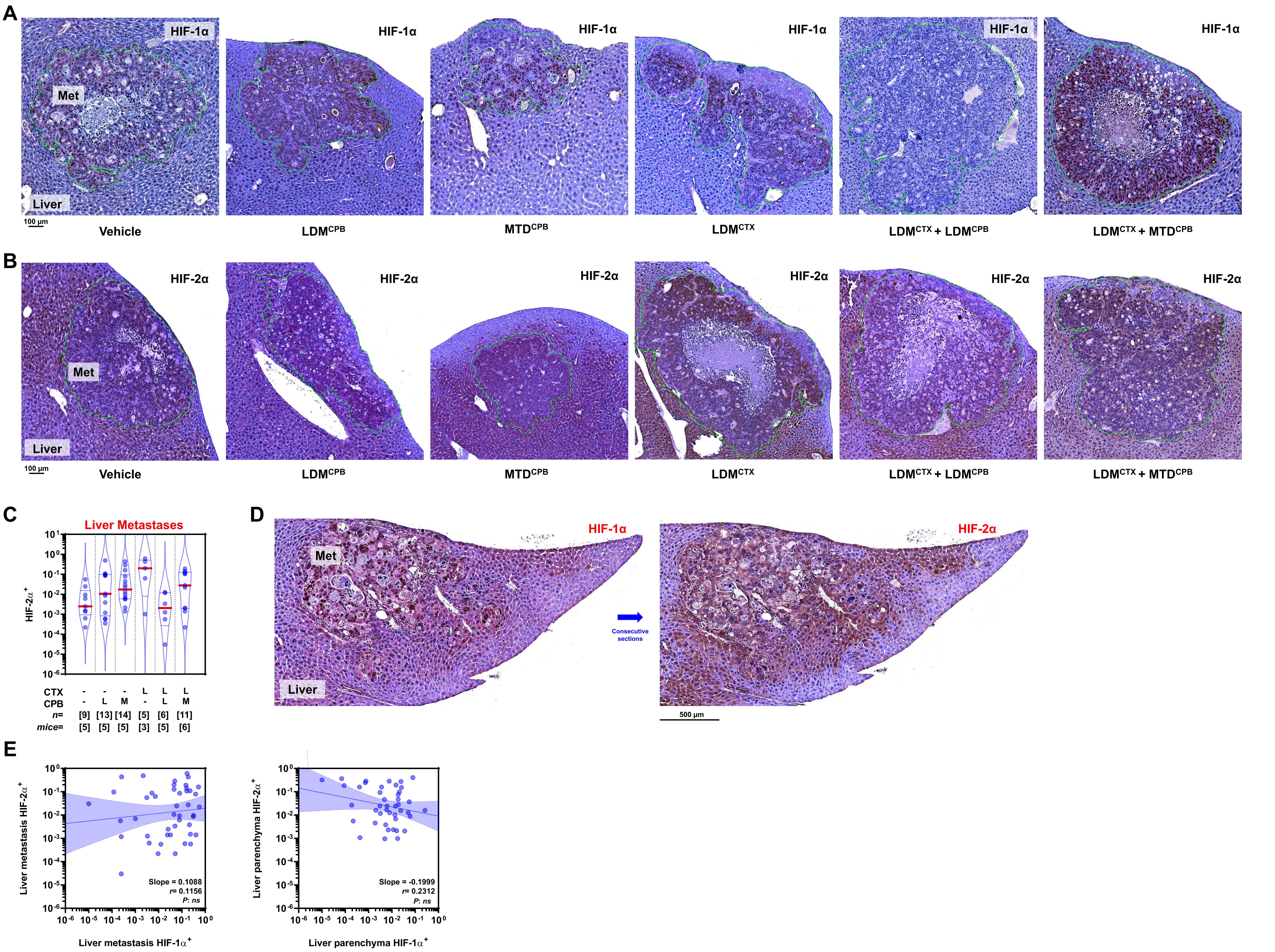

Appendix Figure S4:
HIFα expression in colon cancer liver metastases

Extended image set of Figure 2B showing all experimental groups, including non-statistically significant treatments after automated immunoreactive area quantification.

A, B: HIF-1α (A) or HIF-2α (B) expression in HT29 colon cancer liver metastases. Vehicle, LDM^{CTX} + LDM^{CPB} and LDM^{CTX} + MTD^{CPB} images in Figure S4A correspond to Figure 2B (*left*, *center/left* and *center/right*, respectively).

C: Quantification of HIF-2α expression in HT29 colon cancer liver metastases.
$F_{(5,9)}$= 2.650 and P= 0.0971 (not significant) by one-way Brown-Forsythe ANOVA.

D: Consecutive liver sections (indicated by the *blue arrow*) showing HIF-1α (*left*) or HIF-2α (*right*) expression in HT29 metastases. Images in S4D (*left*) and (*right*) correspond to low-magnification views of Figure 2C and 2D, respectively.

E: Correlation between intra-metastatic and peri-metastatic parenchymal HIF-1α or HIF-2α.

Left: Intra-metastatic HIF-1α *vs* HIF-2α.

$F_{(1,43)}$= 1.290, P= 0.4495 (not significant); slope ≠0 by F-test; n= 45.

Right: Peri-metastatic parenchymatous HIF-1α *vs* HIF-2α.

$F_{(1,41)}$= 2.316, P= 0.1358 (not significant); slope ≠0 by F-test; n= 43.

Data information: Violin plots present 50th (red line), 25th and 75th percentiles (blue lines); Pearson regression line (blue) and 95% CI (shaded blue area) is shown. HIF-1α and HIF-2α are expressed as a fraction of tumoral area. Brackets: n, number of tumors or mice. CPB, capecitabine; CTX, cyclophosphamide; L or LDM, low-dose metronomic; M or MTD, maximum-tolerated dose; *ns*, not significant; *r,* correlation coefficient. *Met*, metastasis.

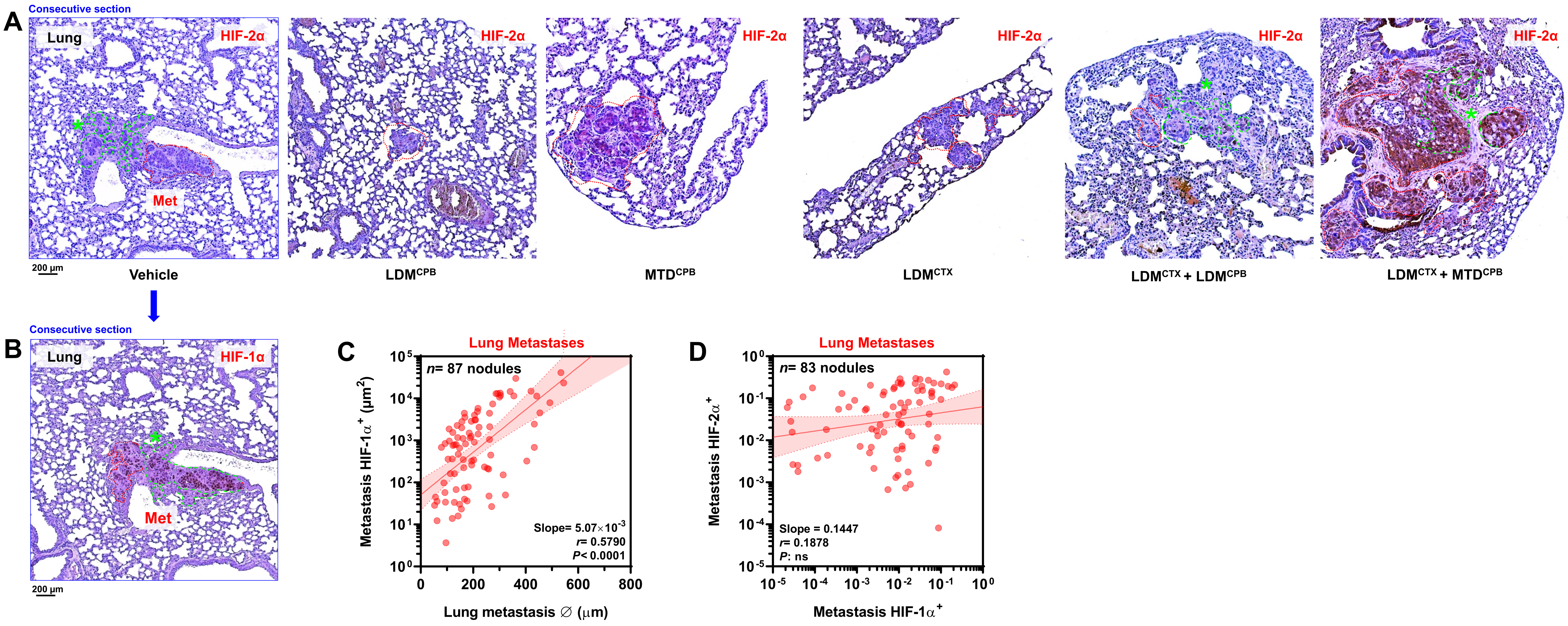

Appendix Figure S5:
Effect of LDM and/or MTD chemotherapy upon HIF-2α in colon cancer metastases to the lungs

Extended image set of Figure EV1B, showing all experimental groups, including non-statistically significant regimens after automated immunoreactive area quantification.

A: HIF-2α levels in HT29 colon cancer metastatic nodules to the lungs.
Green asterisks (*) and green dashed lines in Vehicle, LDM^{CTX} + LDM^{CPB} and LDM^{CTX} + MTD^{CPB} images refer to their respective lung areas depicted at higher magnification in Figure EV1B (*left*, *center/left* and *center/right*, respectively). Vehicle, LDM^{CTX} + LDM^{CPB} and LDM^{CTX} + MTD^{CPB} images in S5A correspond to Figure EV1B (*left*, *center/left* and *center/right*, respectively); *Met*, metastasis.

B: Example of HIF-1α expression in HT29 colon cancer metastatic nodules to the lungs.
Green asterisk (*) and green dashed line correspond to the lung area depicted at higher magnification in Figure EV1D.
Image in S5B corresponds to lower magnification of Figure EV1D.
Blue framed images in Figure S5A and S5B indicate consecutive sections. *Met*, metastasis.

C: Effect of lung metastatic diameter on HIF-1α levels.
Individual nodule diameter and HIF-1α^{+} area (logarithm) were plotted to determine overall correlation.
Regression line (red) and 95% CI (shaded red area) are shown.
$F_{(1,85)}$= 42.86 and P< 0.0001 by F-test. *n*, number of nodules.

D: Correlation between lung intra-metastatic HIF-1α^{+} and HIF-2α^{+} fractional areas.
Regression line (red) and 95% CI (shaded red area) are shown.
$F_{(1,81)}$= 2.963 and P= 0.089 (not significant) by F-test. *n*, number of nodules.

Data information: Green+red and red dashed lines in Figures S5A and S5B encircle the histological limit between metastatic nodules and their surrounding normal lung parenchyma. CPB, capecitabine; CTX, cyclophosphamide; LDM, low-dose metronomic; MTD, maximum-tolerated dose; *ns*, not significant; *r*, Pearson correlation coefficient.

Appendix Figure S6

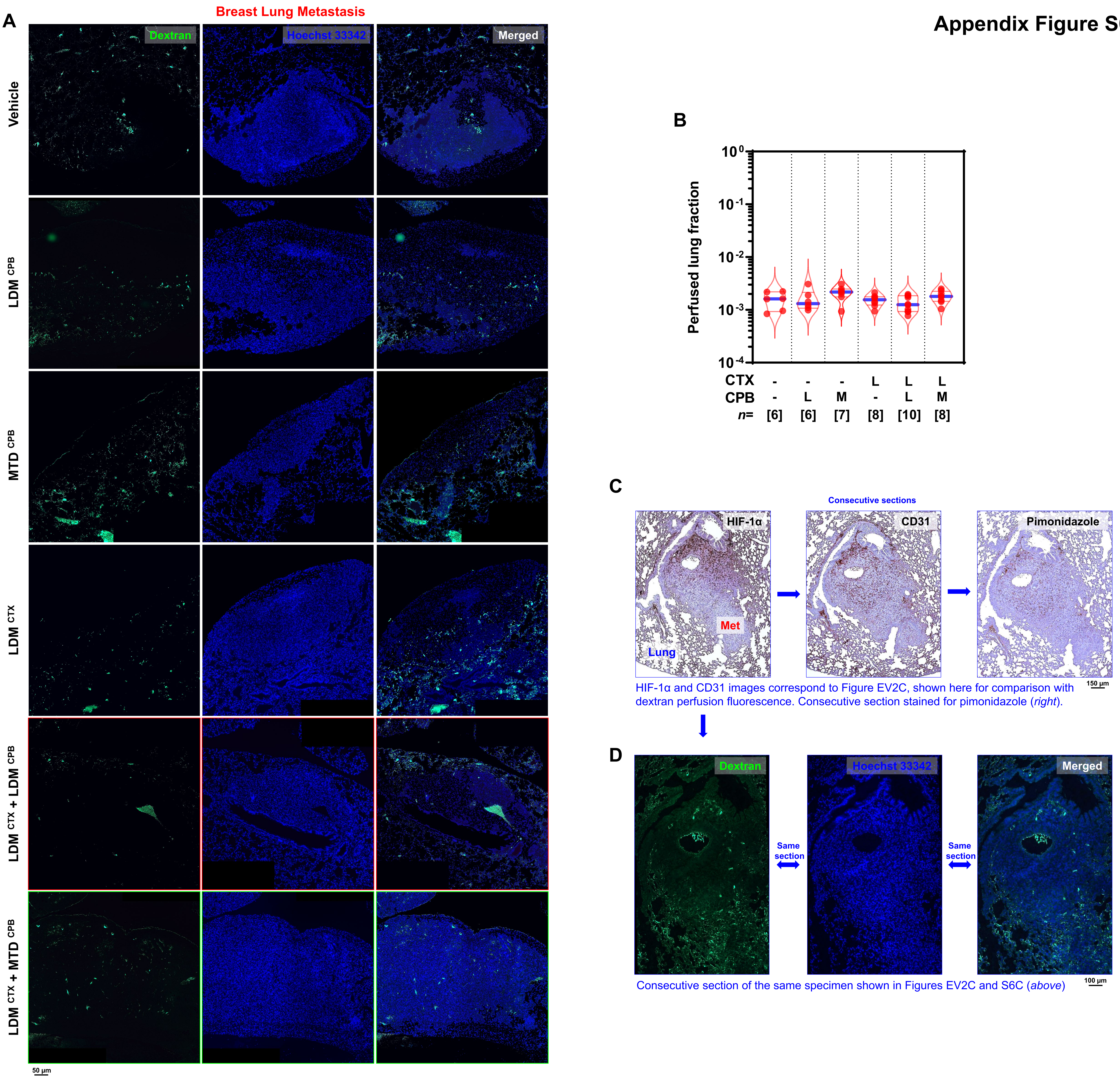

Appendix Figure S6:
Lung parenchymal perfusion in metastatic EMT6-CDDP breast cancer

A: Immunofluorescence imaging of fluorescein-labeled dextran, depicting perfused vessels within lung sections.

Low magnification images of breast metastatic nodules to the lungs. Perfused vessel *lumina* are labeled in green (dextran, *left column*); nuclei are counterstained blue (Hoechst 33342, *middle column*). Merged images (*right column*) reveal mostly peri-metastatic perfusion.

B: Quantification of lung perfusion images. Dextran-perfused pixels were quantified and normalized against total lung section areas. $F_{(5,39)}$=1.591 and P= 0.1853 (not significant) by Brown-Forsythe one-way ANOVA. *n*, number of perfused mice (or lung pairs).

C: Consecutive sections showing the localization of intra-metastatic HIF-1α^{+}, CD31^{+} vessels and hypoxia (pimonidazole^{+}) in an example of a breast metastasis to the lung. *Met*, metastasis.

D: Perfusion image of a consecutive section in the same metastatic nodule shown in (**C**).

Data information: Violin plots present 50th (blue line), 25th and 75th percentiles (red lines); CPB, capecitabine; CTX, cyclophosphamide; L or LDM, low-dose metronomic; M or MTD, maximum-tolerated dose. Red-framed images correspond to consecutive sections of samples stained for pimonidazole and HIF-1α in Figures 3B and 3C; similarly, green-framed images correspond to a consecutive pimonidazole-stained section from Figure 3B. Black boxes ("zig-zag") in some topograms (Figures S6A and S6D) result from automated stitching of digital microphotograph mosaics, wherein non-square matrices of input images reveal the underlying black digital canvas. Blue frames indicate consecutive sections (Figures S6C and S6D).

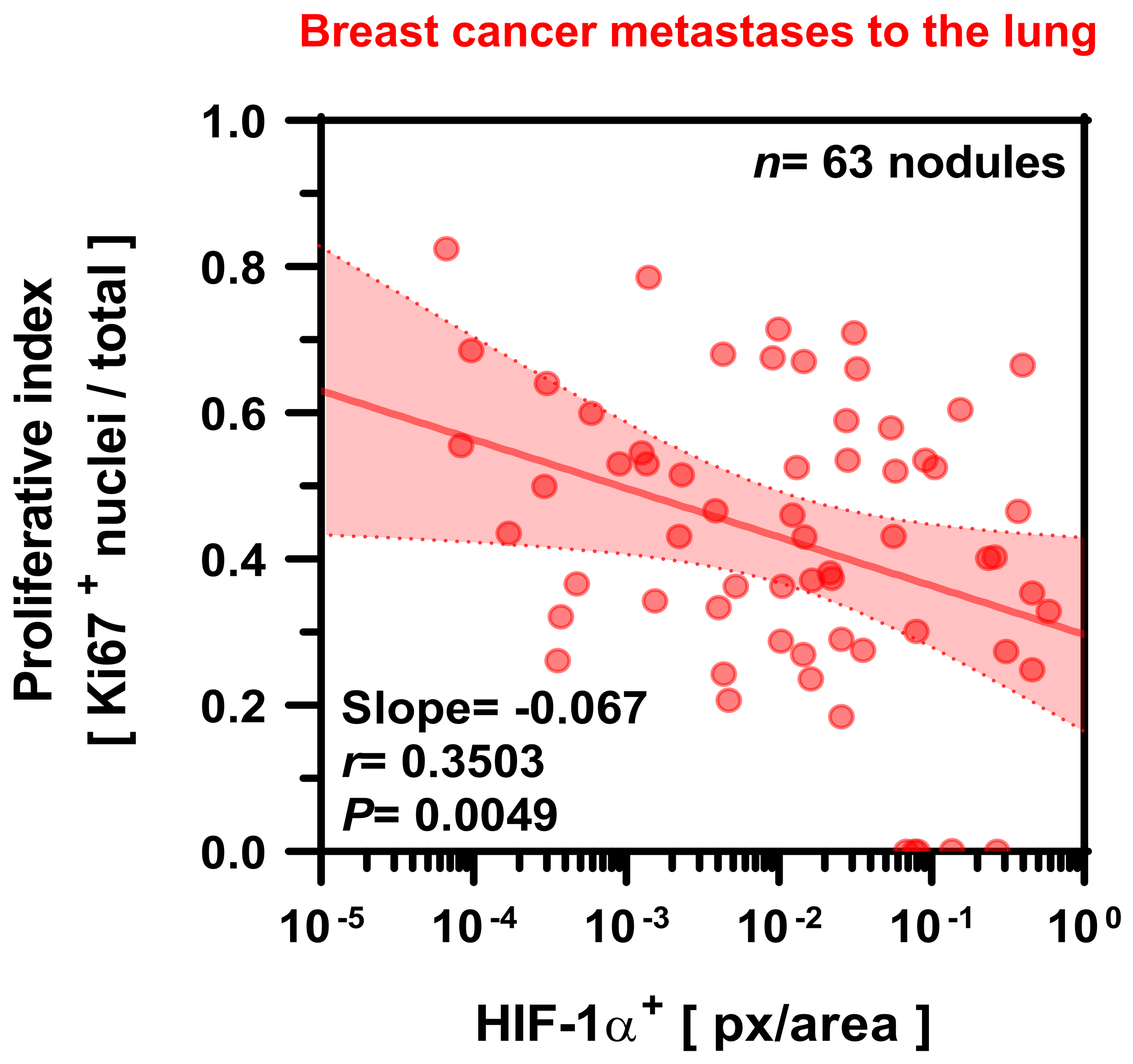

Appendix Figure S7:
HIF-1α-dependent proliferative index in breast cancer metastatic nodules to the lungs

Correlation between intra-metastatic HIF-1α levels and Ki67 proliferative indexes.

$F_{(1,61)}$= 8.528, P= 0.0049; slope ≠ 0 by F-test; n= 63.

Data information: Pearson regression line (red) and 95% CI (shaded red area) is shown.

HIF-1α^{+} signal is expressed as a fraction of tumoral sectional area. *n*, number of lung metastases; *r*, correlation coefficient.

Appendix Table S1 – List of antibodies

Reagent	Cat. #	Vendor	Working Dilution
anti-HIF-1 alpha	NB100-105	Novus Biologicals	1:50
anti-HIF-2 alpha	NB100-122	Novus Biologicals	1:200
anti-CD31	sc-1506	Santa Cruz Biotechnology	1:500
anti-CA9	AF2188	R&D Systems	1:150
anti-Ki67	VP-K451	Vector Laboratories	1:1,000
anti-pimonidazole mouse IgG1 MAb1	HP1	Hypoxyprobe	1:50
