# Supplementary material for: Metronomic chemotherapy offsets HIFα induction upon maximum‐tolerated dose in metastatic cancers
Source: EMBO Mol Med. 2020 Jul 20;12(9):e11416. doi: 10.15252/emmm.201911416 (PMC7507002; doi:10.15252/emmm.201911416)
Supplement: Supplementary file 2 — Expanded View Figures PDF [file EMMM-12-e11416-s002.pdf]

Expanded View Figures

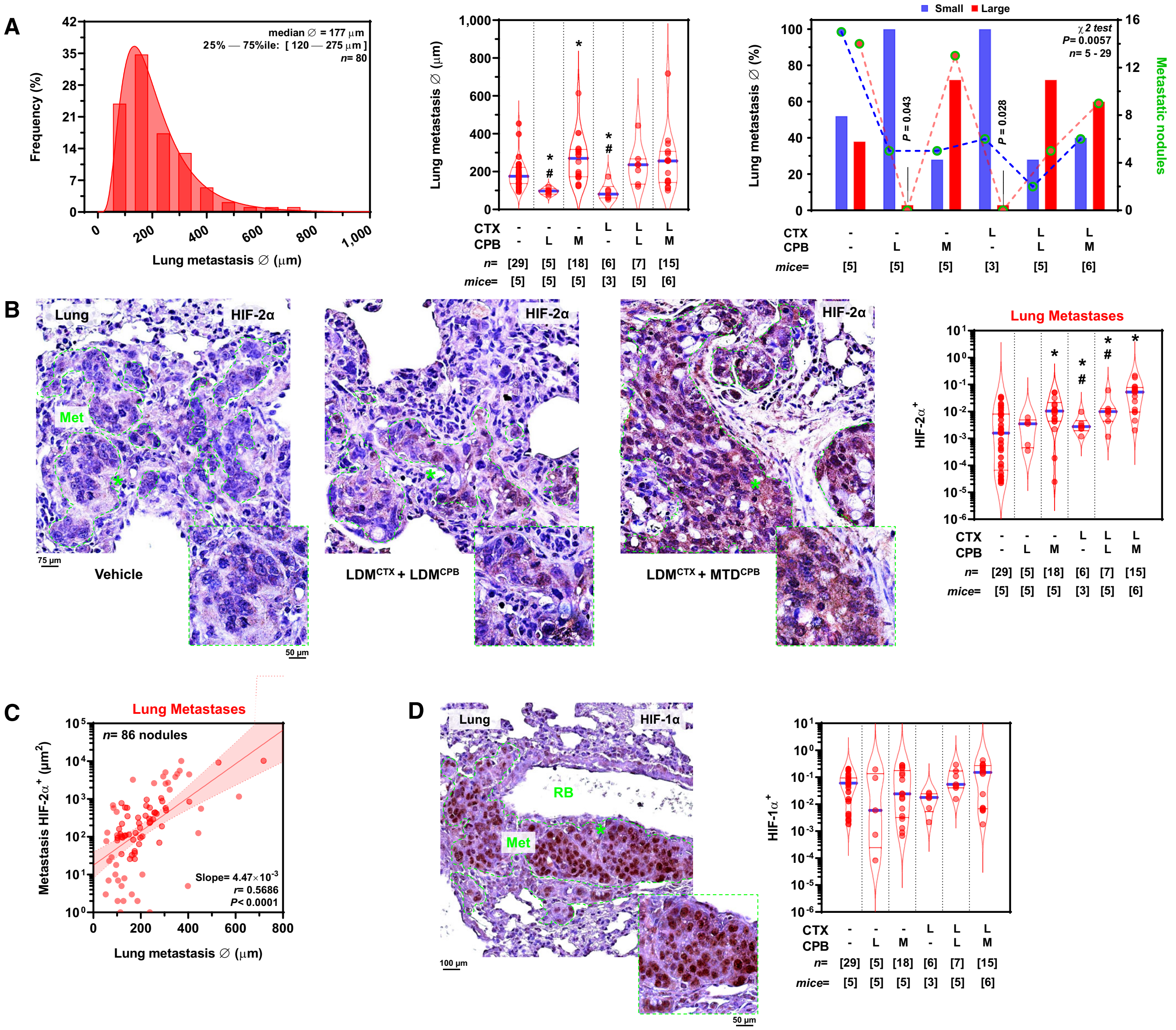

Figure EV1.

◀ **Figure EV1. LDM chemotherapy offsets HIF-2α levels in colon cancer metastases to the lung.**

A Effect of LDM and MTD chemotherapy on HT29 lung metastatic nodule size. *Left*: Histogram of cross-sectional metastatic diameter (Ø). *Middle*: Metastatic diameter (Ø) by chemotherapy regimen. $F_{5,43}$ = 9.066 and $P < 0.0001$ for overall treatment by Brown–Forsythe ANOVA; *$P < 0.05$ versus vehicle; $^{\#}P < 0.01$ versus LDM^{CTX} + MTD^{CPB} by Benjamini, Krieger, and Yekutieli *post hoc* test. *Right*: Dichotomized metastatic size at median diameter of vehicle-treated tumors. Lung nodule size was classified as small (blue) or large (red). Individual nodule counts per group and size category (small/large) are plotted on the right ordinate. χ^2 (df = 5) = 16.44, $P = 0.0057$ for overall effects on size; χ^2 (df = 1) = 4.10, $P = 0.043$ LDM^{CPB} versus vehicle; χ^2 (df = 2) = 7.00, $P = 0.028$ LDM^{CTX} versus vehicle.

B HIF-2α levels in HT29 lung metastatic nodules. *Left*: vehicle-treated controls. *Middle/left*: LDM doublet cyclophosphamide + capecitabine (LDM^{CTX} + LDM^{CPB}). *Middle/right*: LDM cyclophosphamide + MTD capecitabine (LDM^{CTX} + MTD^{CPB}). *Right*: Automatic quantification of the effect of monotherapies or doublet regimens on HIF-2α^{+} areas in individual metastatic nodules. $F_{5,64}$ = 12.38 and $P < 0.0001$ for overall treatment by Brown–Forsythe ANOVA; *$P < 0.01$ versus vehicle; $^{\#}P < 0.05$ versus LDM^{CTX} + MTD^{CPB} by Benjamini, Krieger, and Yekutieli *post hoc* test.

C Effect of lung metastatic diameter on HIF-2α levels. Individual nodule diameter and HIF-2α^{+} area (logarithm). Regression line (red) and 95% CI (shaded red area) are shown. $F_{1,84}$ = 40.18 and $P < 0.0001$; slope $\neq 0$ by *F*-test.

D HIF-1α levels in HT29 lung metastatic nodules. *Left*: Example of HIF-1α expression in a para-bronchiolar nodule. *RB*, respiratory bronchiole showing lysed intralumenal erythrocytes. Low power magnification image can be found in Appendix Fig S5B. *Right*: Automatic quantification of the effect of monotherapies or doublet regimens upon intra-metastatic HIF-1α^{+} area. $F_{5,14}$ = 1.670 and $P = 0.2053$ (not significant) for overall treatment by Brown–Forsythe ANOVA.

Data information: Violin plots present 50th (blue line), 25th, and 75th percentiles (red lines); numbers in brackets indicate number of nodules (*n*) or animals (mice). *L*, low-dose metronomic; *M*, maximum-tolerated dose; *Met*, metastasis; *r*, correlation coefficient. Dashed green lines encircle the histological limit between metastatic nodules and surrounding normal lung parenchymae. *Insets*, high-magnification images of regions marked with asterisks (* in all microphotographs). Low power magnification images of all experimental conditions of B can be found in Appendix Fig S5A.

 © 2020 The Authors

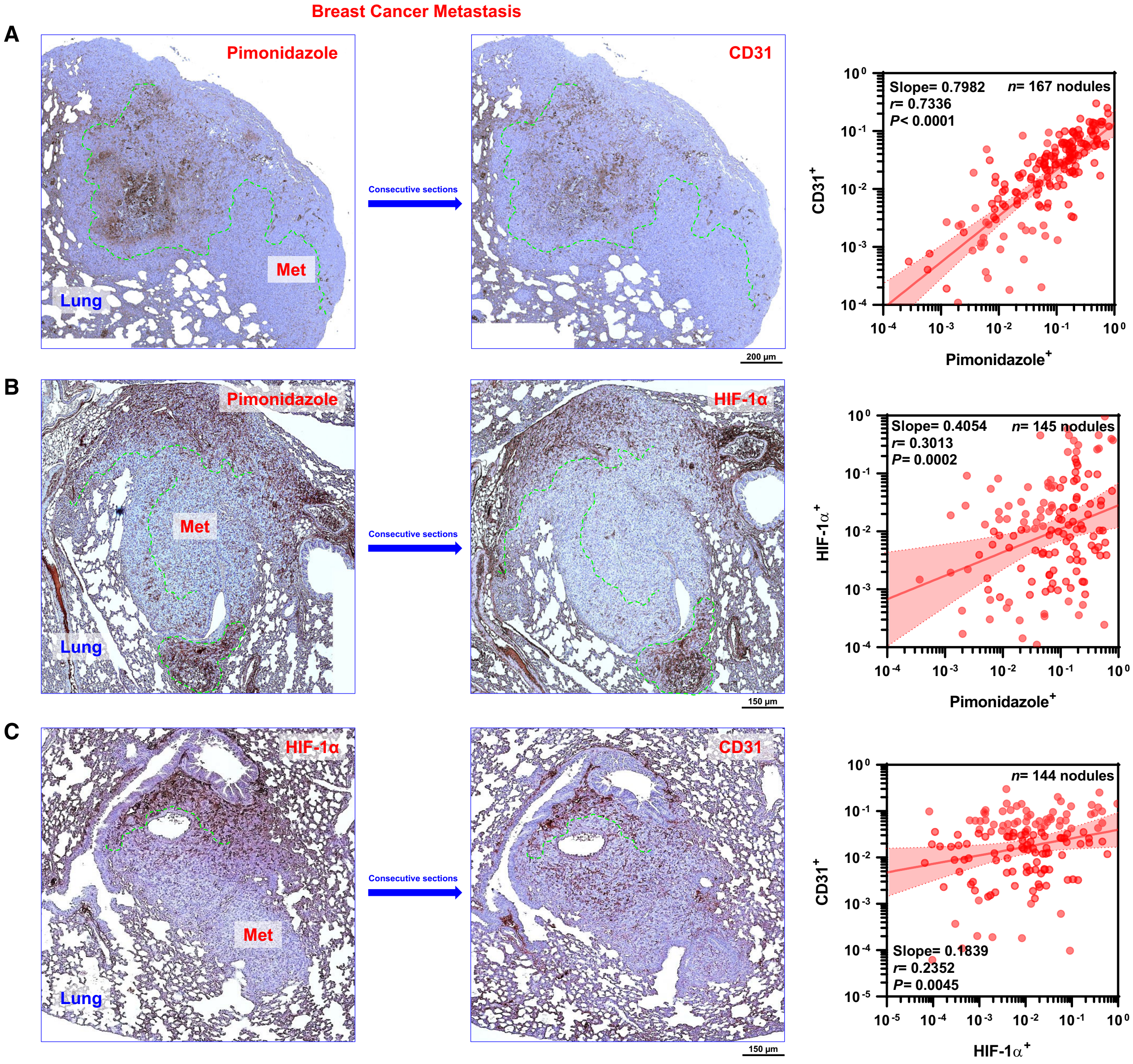

Figure EV2. Relationship among hypoxia, HIF-1α, and microvessel density in breast cancer metastases to the lung.

A Intra-metastatic hypoxia (pimonidazole) and microvessel density (CD31). *Left* and *middle*: Consecutive sections showing pimonidazole (*left*) and CD31 (*middle*) immunoreactivities. *Right*: Correlation between intra-metastatic hypoxia (pimonidazole) and microvessel density (CD31). Points represent median values per nodule. $F_{1,165}$ = 192.2 and $P < 0.0001$, slope ≠ 0 by *F*-test.

B Intra-metastatic hypoxia (pimonidazole) and HIF-1α levels. *Left* and *middle*: Consecutive sections showing pimonidazole (*left*) and HIF-1α (*middle*) immunoreactivities. *Right*: Correlation between intra-metastatic hypoxia (pimonidazole) and HIF-1α. Points represent median values per nodule. $F_{1,143}$ = 14.3 and P = 0.0002, slope ≠ 0 by *F*-test.

C HIF-1α levels and microvessel density (CD31). *Left* and *middle*: Consecutive sections showing HIF-1α (*left*) and CD31 (*middle*) immunoreactivities. *Right:* Correlation between HIF-1α and CD31. Points represent median values per nodule. $F_{1,142}$ = 8.313 and P = 0.0045, slope ≠ 0 by *F*-test.

Data information: *Pearson* regression line (red) and 95% CI (shaded red area) are indicated. Immunoreactivities are expressed as fractions of nodular areas. *Met*, metastasis; *r*, correlation coefficient. Dashed green lines demarcate corresponding intra-metastatic areas of immunoreactivity among pimonidazole, CD31, and HIF-1α in consecutive histological sections from example specimens (blue frames/arrows).

© 2020 The Authors
