# Supplementary figures and images for: Metronomic chemotherapy offsets HIFα induction upon maximum‐tolerated dose in metastatic cancers
Source: EMBO Mol Med. 2020 Jul 20;12(9):e11416. doi: 10.15252/emmm.201911416 (PMC7507002; doi:10.15252/emmm.201911416)

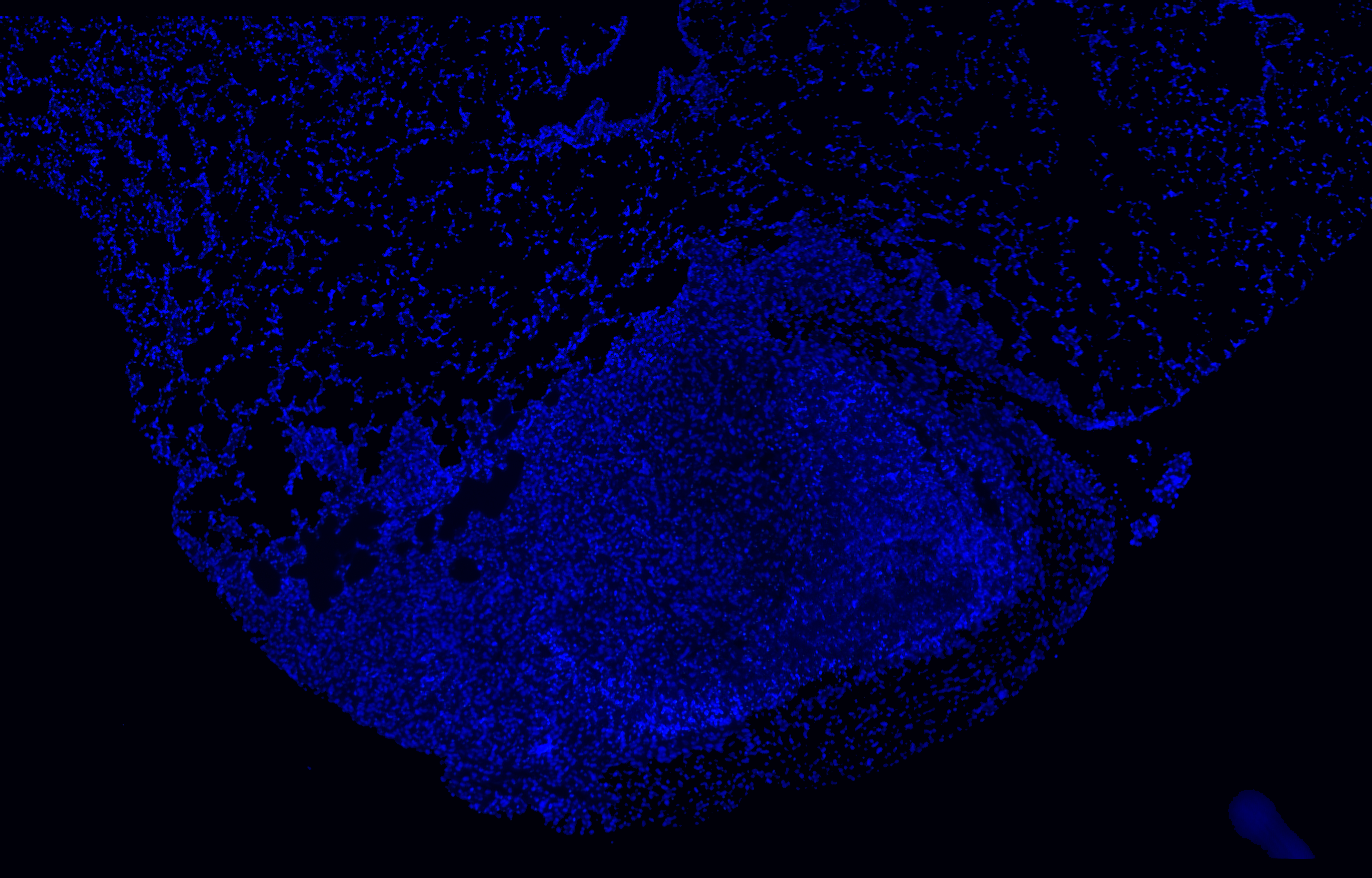

Supplement: Supplementary file 3 — Source Data for Appendix [file EMMM-12-e11416-s004.zip › Figure Appendix S6A/S6A_#1_Vehicle/Dapi.tif]

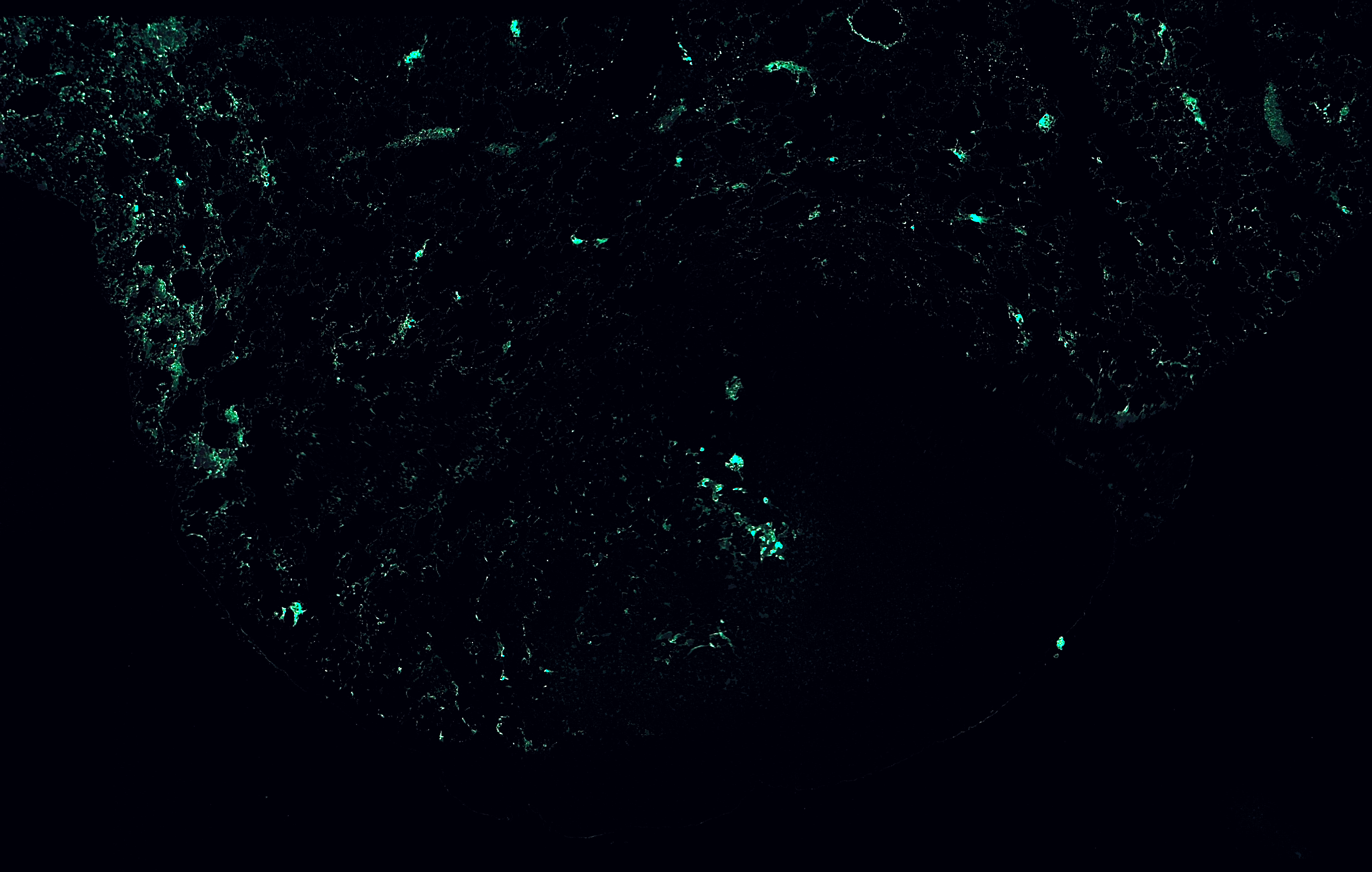

Supplement: Supplementary file 3 — Source Data for Appendix [file EMMM-12-e11416-s004.zip › Figure Appendix S6A/S6A_#1_Vehicle/Dextran.tif]

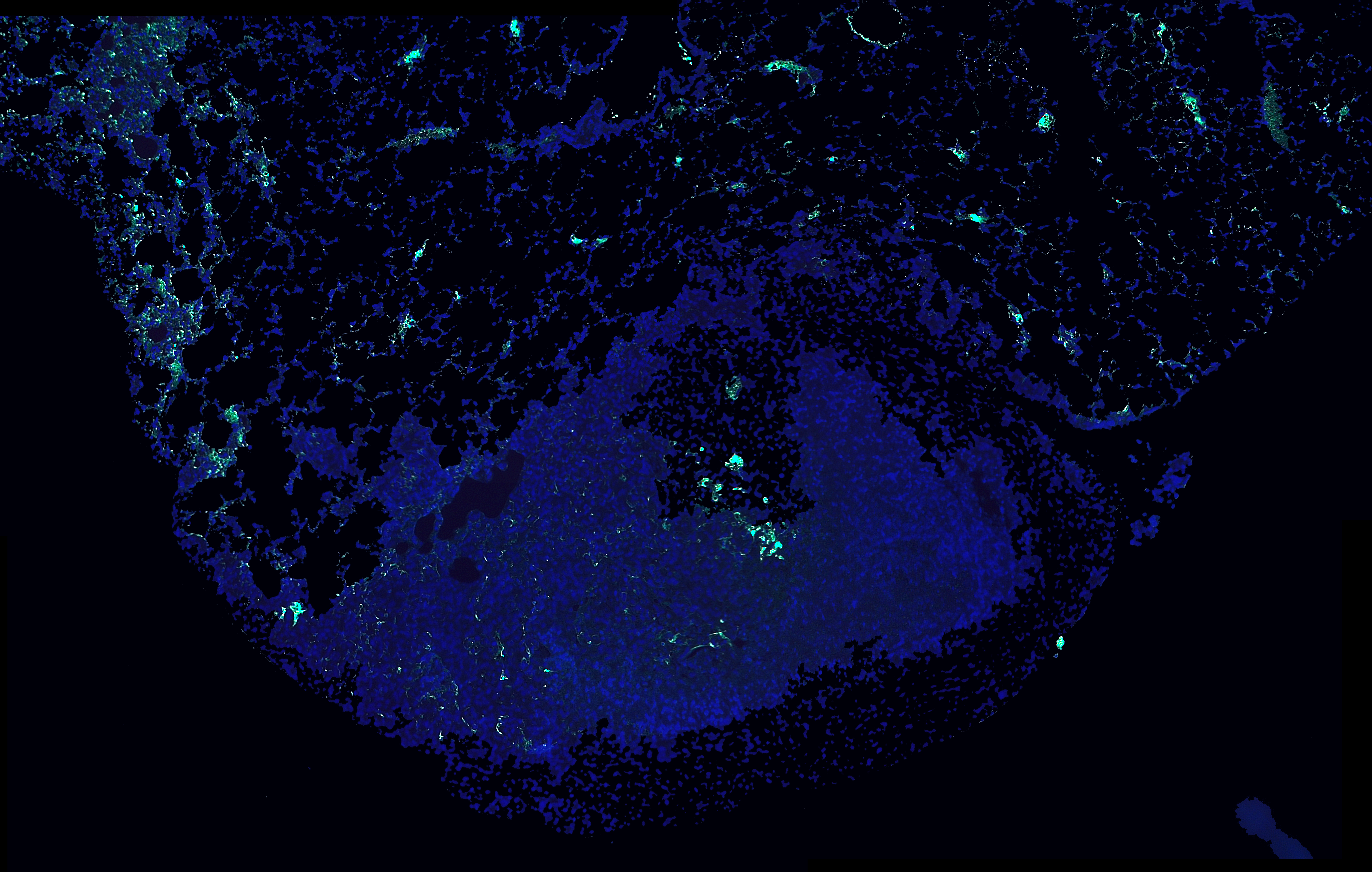

Supplement: Supplementary file 3 — Source Data for Appendix [file EMMM-12-e11416-s004.zip › Figure Appendix S6A/S6A_#1_Vehicle/Merged.tif]

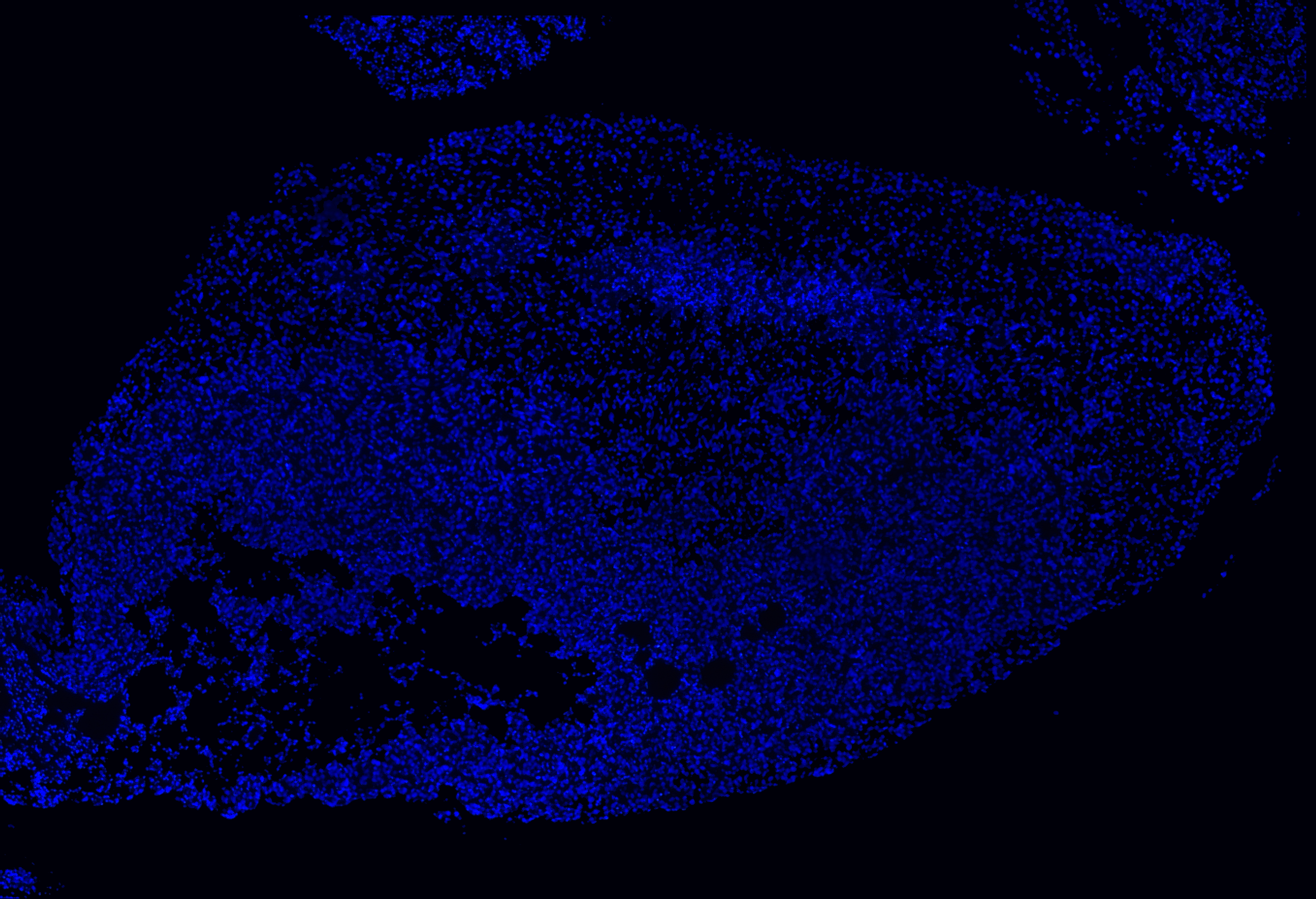

Supplement: Supplementary file 3 — Source Data for Appendix [file EMMM-12-e11416-s004.zip › Figure Appendix S6A/S6A_#2_LDM_CPB/Dapi.tif]

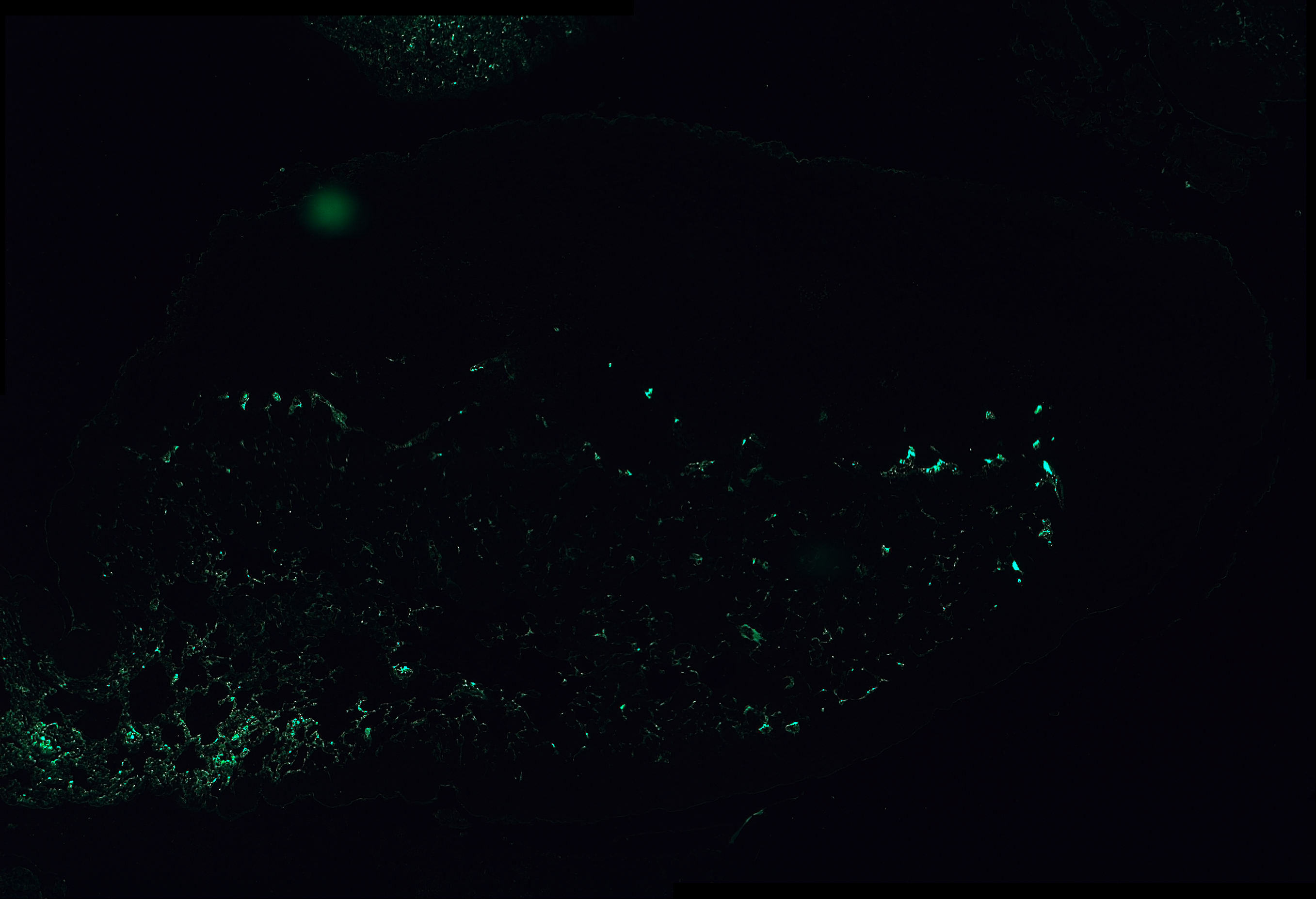

Supplement: Supplementary file 3 — Source Data for Appendix [file EMMM-12-e11416-s004.zip › Figure Appendix S6A/S6A_#2_LDM_CPB/Dextran.tif]

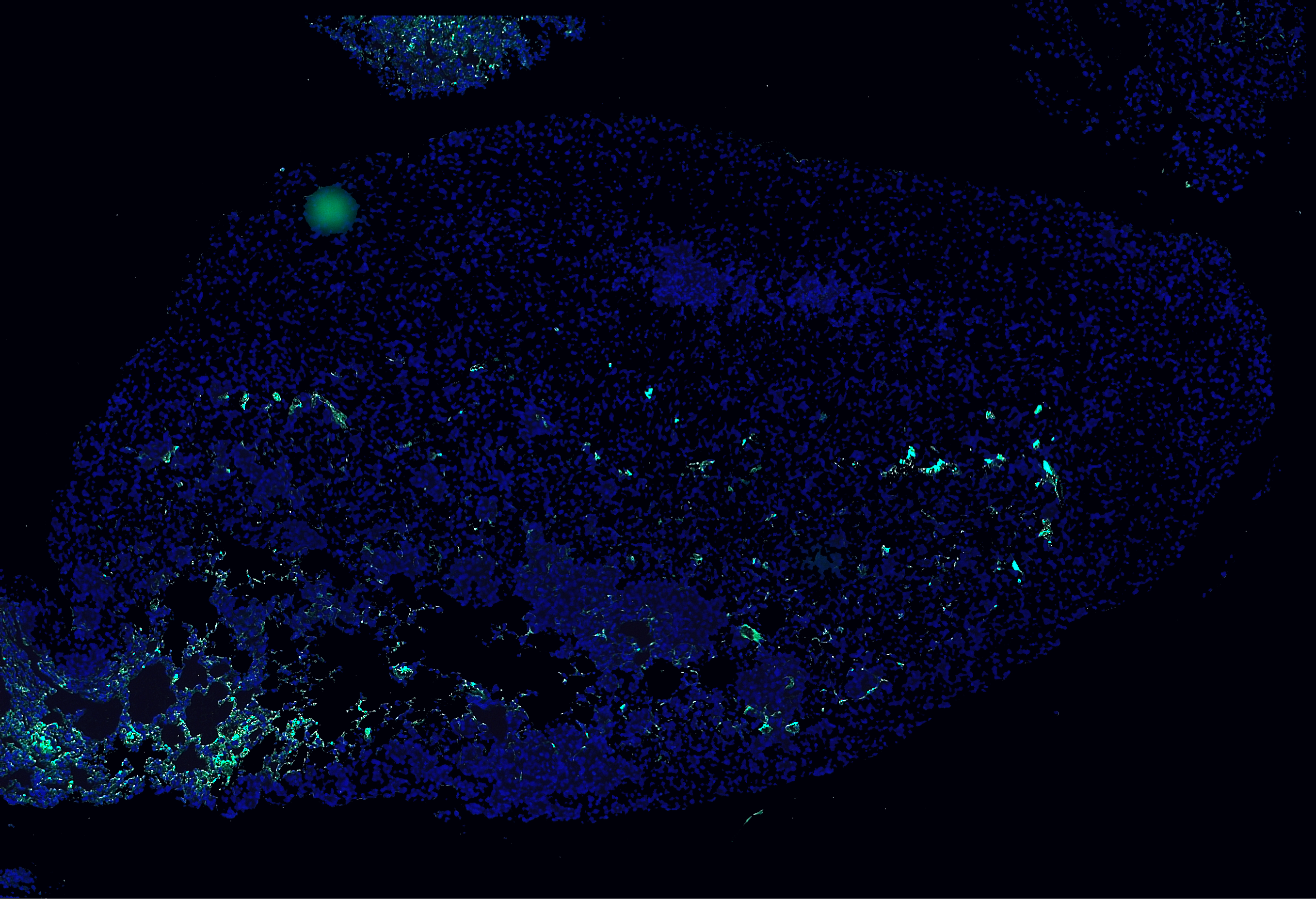

Supplement: Supplementary file 3 — Source Data for Appendix [file EMMM-12-e11416-s004.zip › Figure Appendix S6A/S6A_#2_LDM_CPB/Merged.tif]

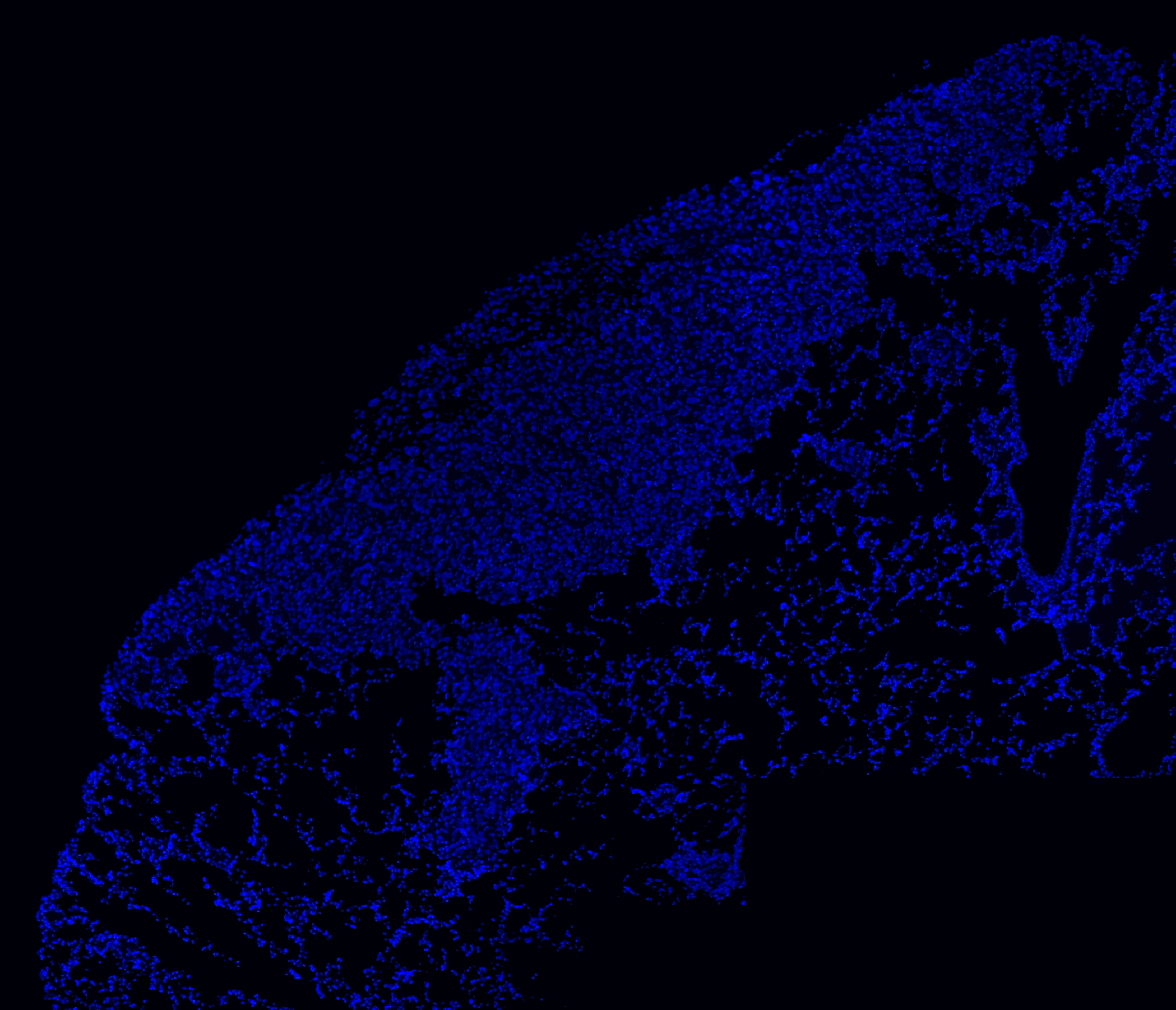

Supplement: Supplementary file 3 — Source Data for Appendix [file EMMM-12-e11416-s004.zip › Figure Appendix S6A/S6A_#3_MTD_CPB/Dapi.tif]

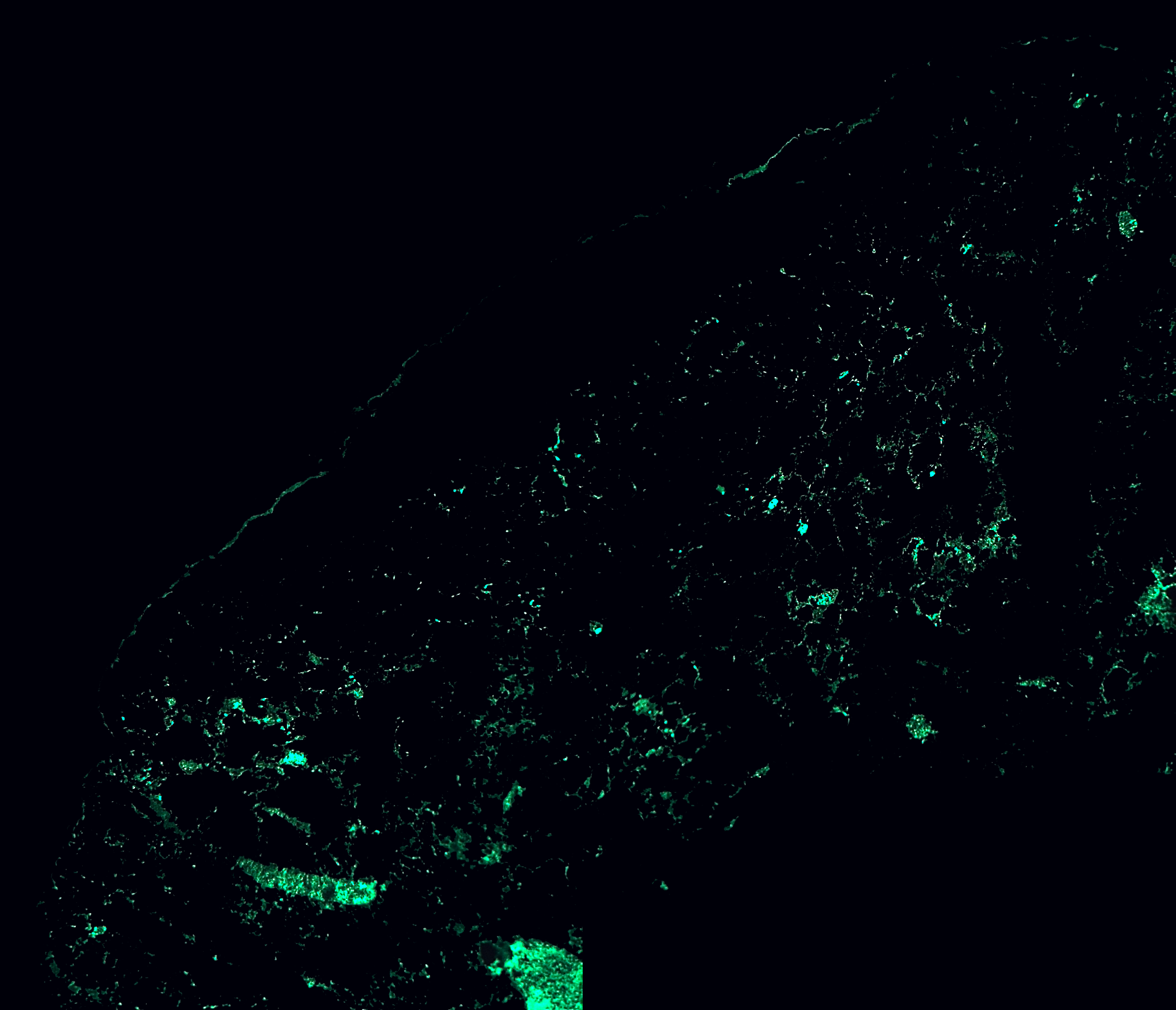

Supplement: Supplementary file 3 — Source Data for Appendix [file EMMM-12-e11416-s004.zip › Figure Appendix S6A/S6A_#3_MTD_CPB/Dextran.tif]

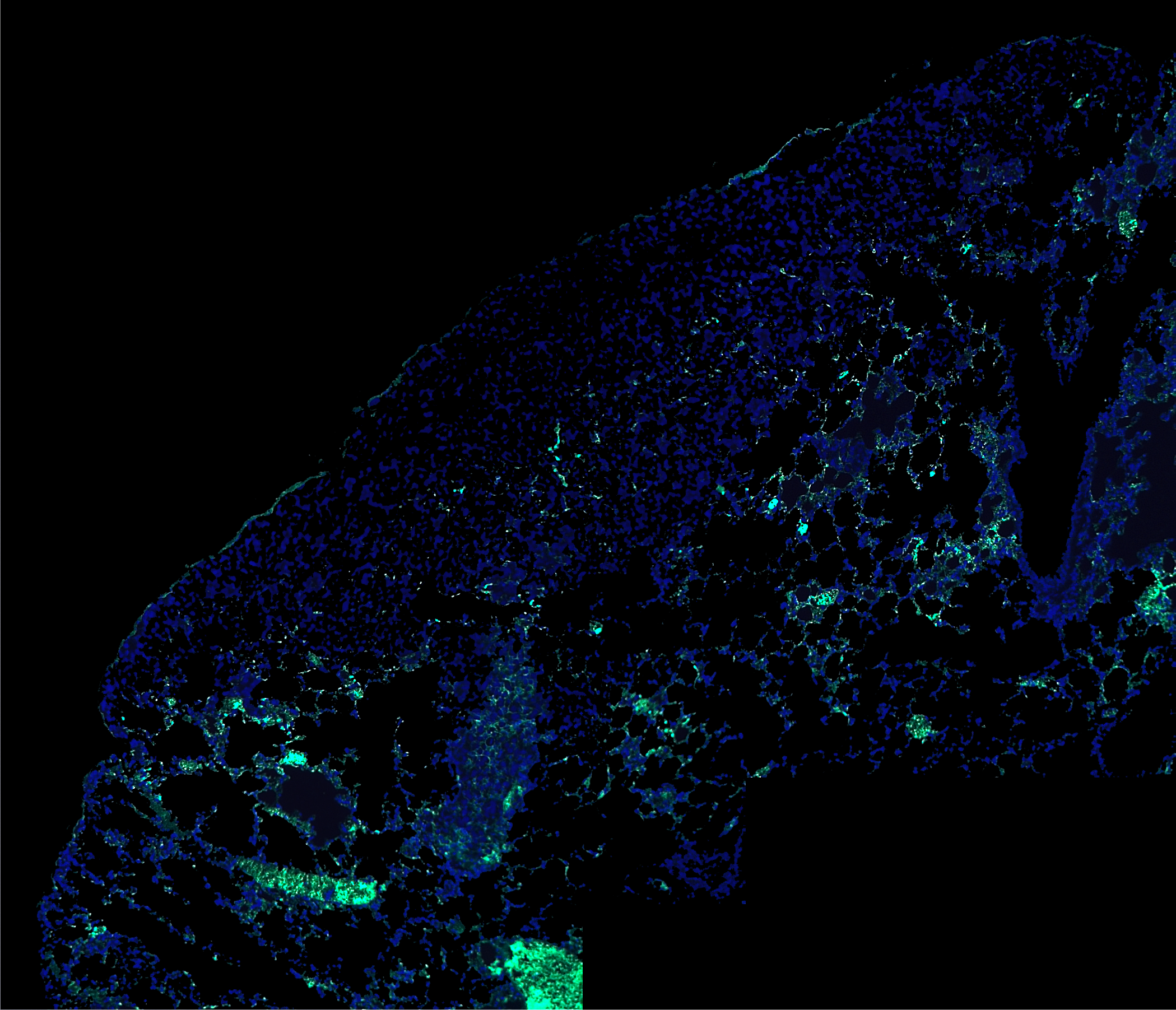

Supplement: Supplementary file 3 — Source Data for Appendix [file EMMM-12-e11416-s004.zip › Figure Appendix S6A/S6A_#3_MTD_CPB/Merged.tif]

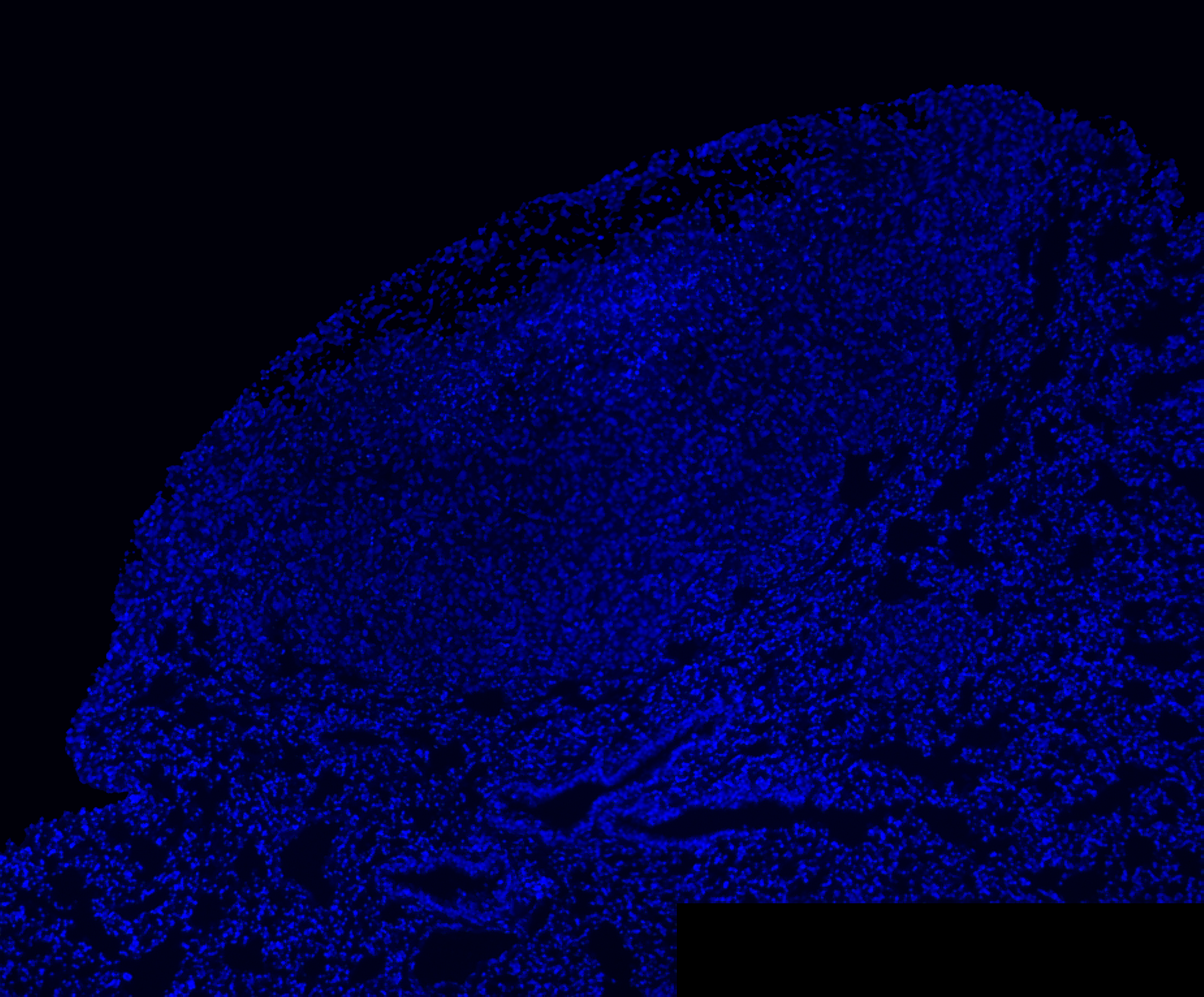

Supplement: Supplementary file 3 — Source Data for Appendix [file EMMM-12-e11416-s004.zip › Figure Appendix S6A/S6A_#4_LDM_CTX/Dapi.tif]

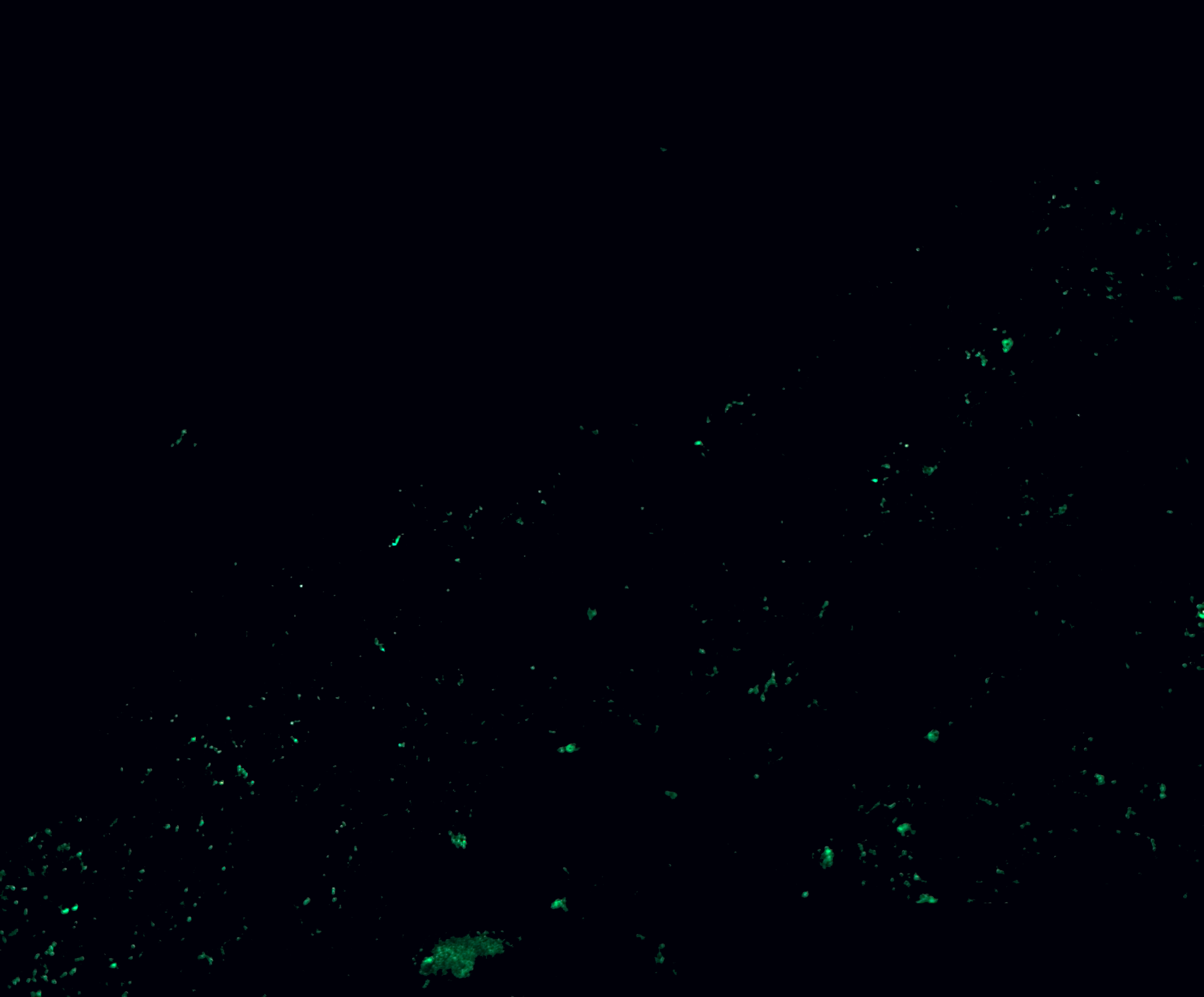

Supplement: Supplementary file 3 — Source Data for Appendix [file EMMM-12-e11416-s004.zip › Figure Appendix S6A/S6A_#4_LDM_CTX/Dextran.tif]

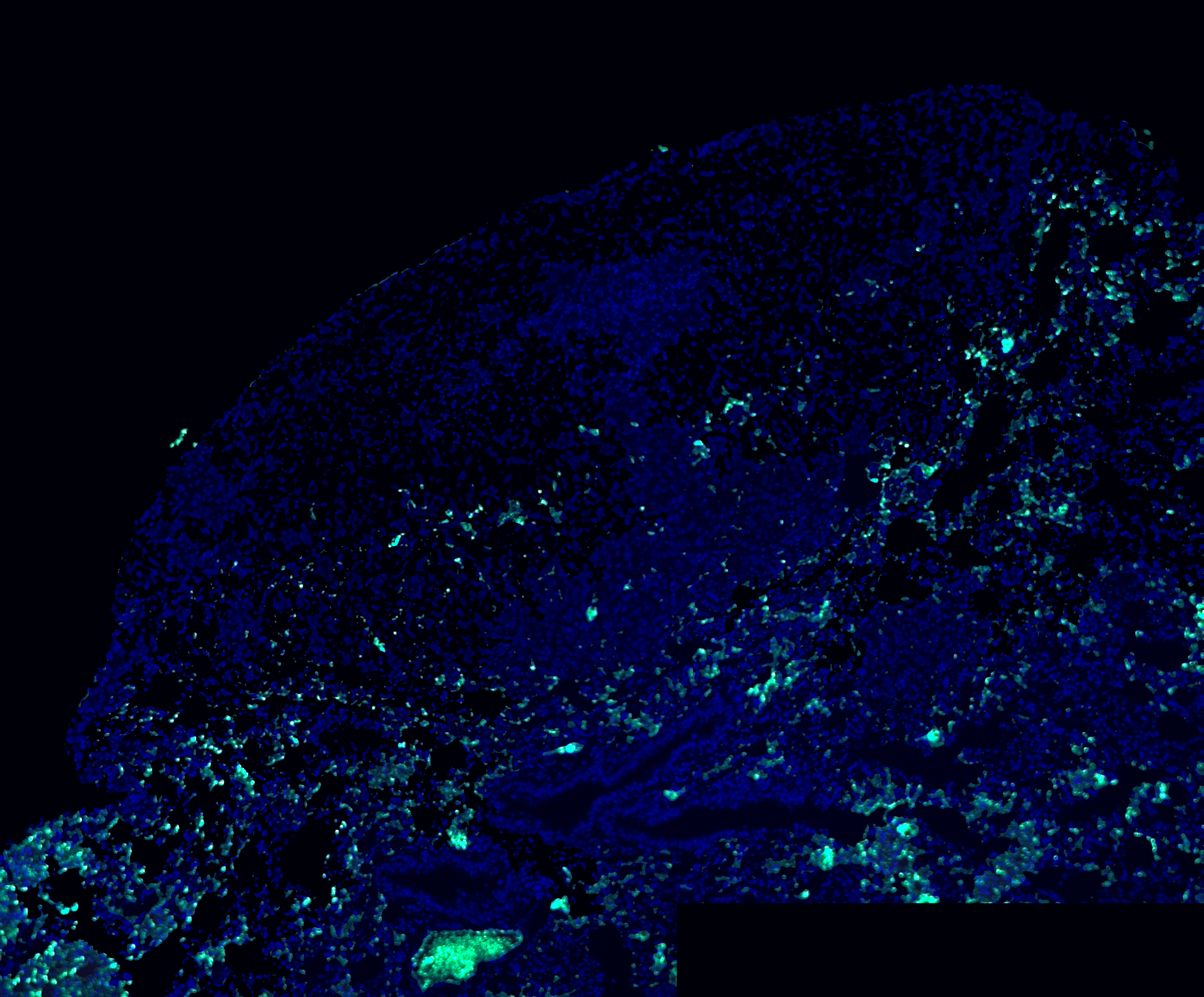

Supplement: Supplementary file 3 — Source Data for Appendix [file EMMM-12-e11416-s004.zip › Figure Appendix S6A/S6A_#4_LDM_CTX/Merged.tif]

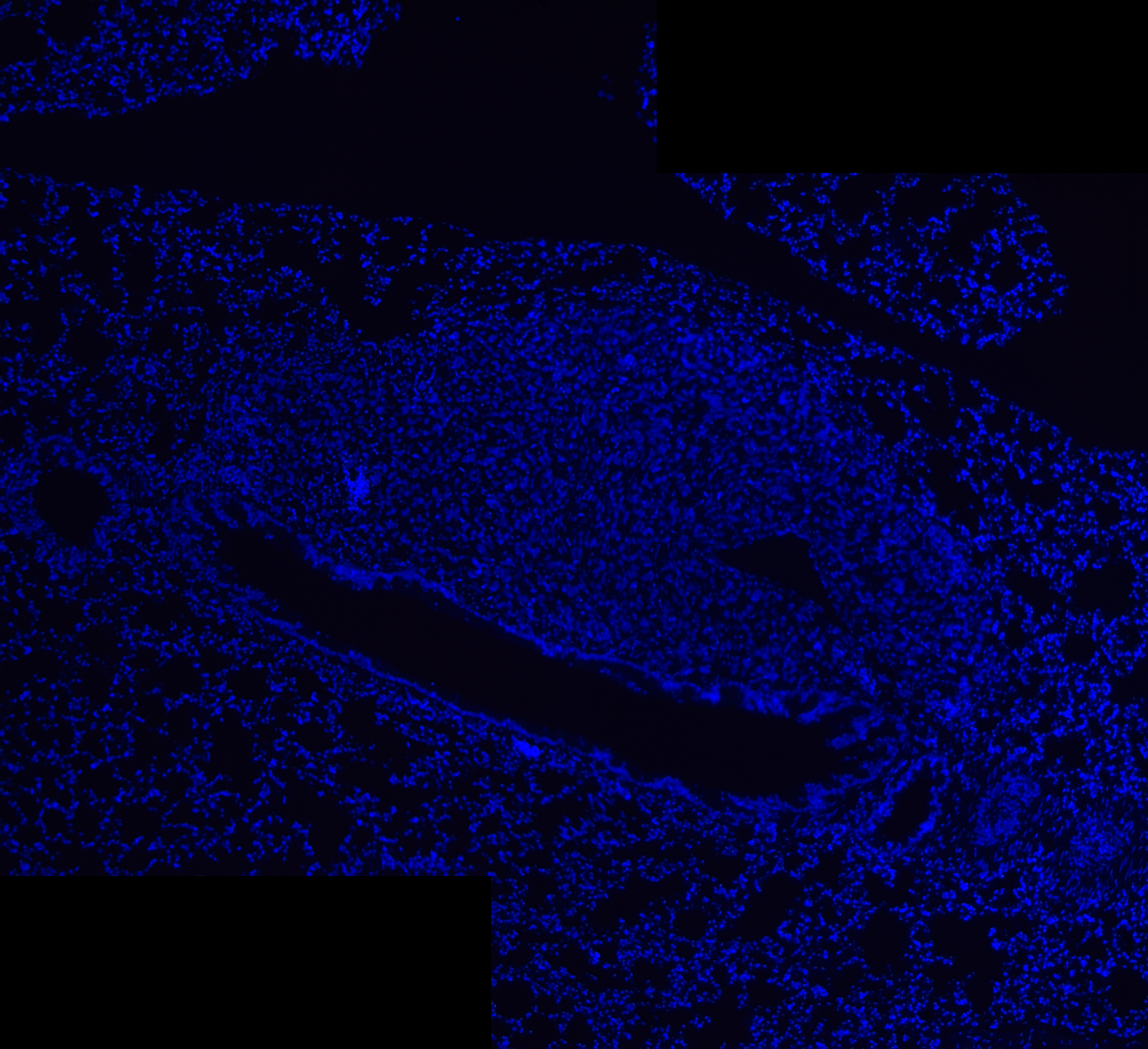

Supplement: Supplementary file 3 — Source Data for Appendix [file EMMM-12-e11416-s004.zip › Figure Appendix S6A/S6A_#5_LDM CTX_+_LDM_CPB/Dapi.tif]

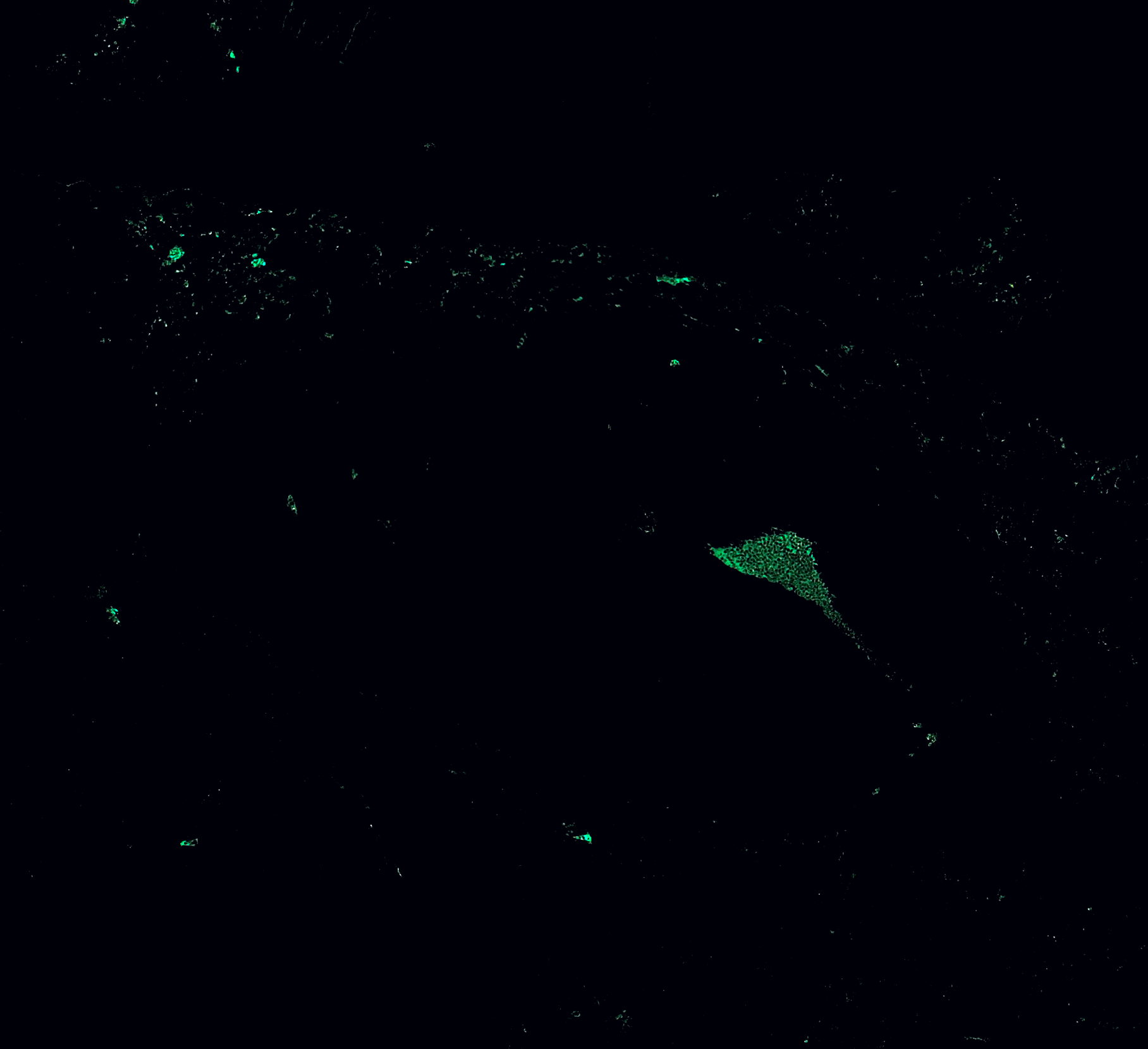

Supplement: Supplementary file 3 — Source Data for Appendix [file EMMM-12-e11416-s004.zip › Figure Appendix S6A/S6A_#5_LDM CTX_+_LDM_CPB/Dextran.tif]

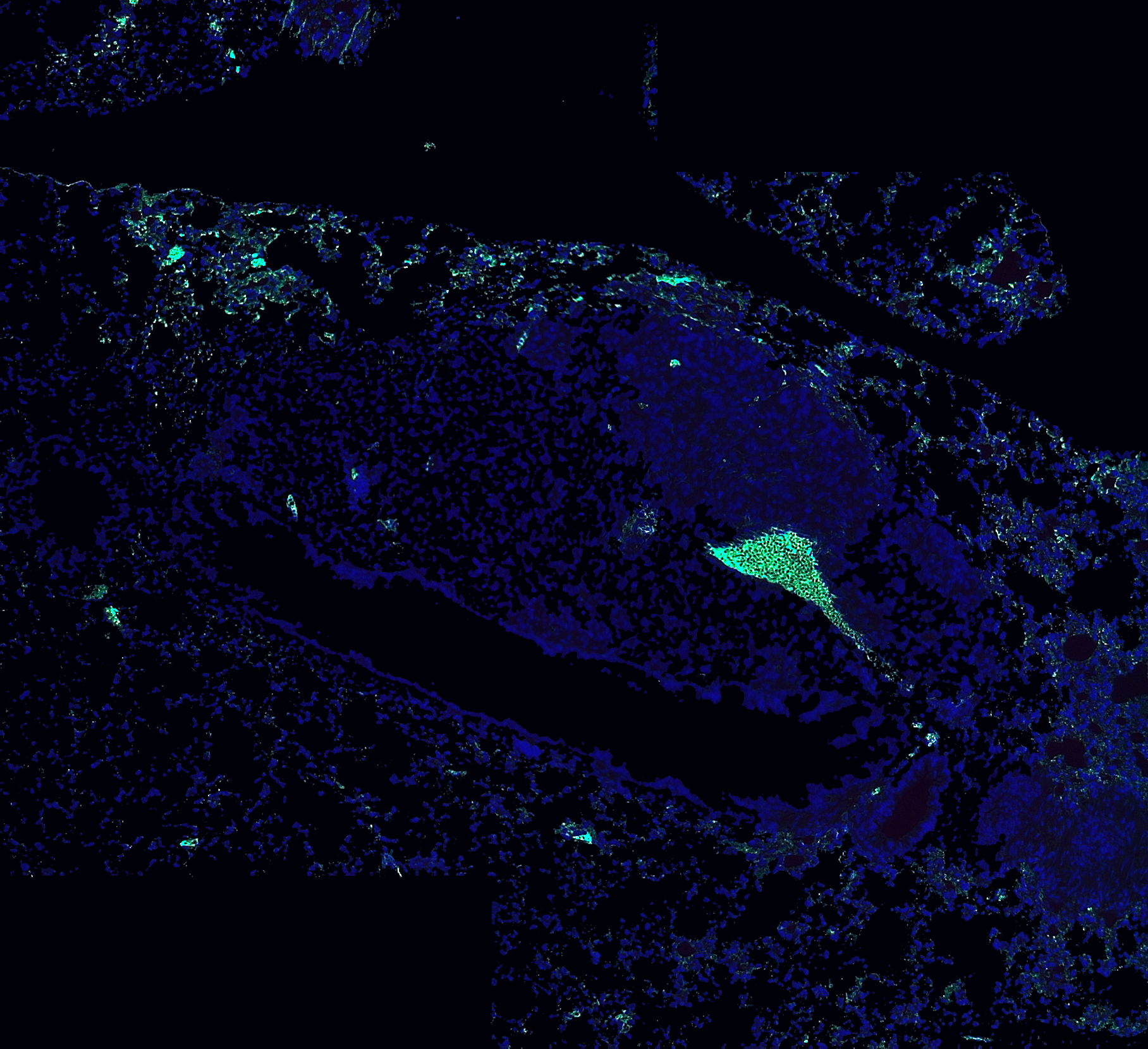

Supplement: Supplementary file 3 — Source Data for Appendix [file EMMM-12-e11416-s004.zip › Figure Appendix S6A/S6A_#5_LDM CTX_+_LDM_CPB/Merged.tif]

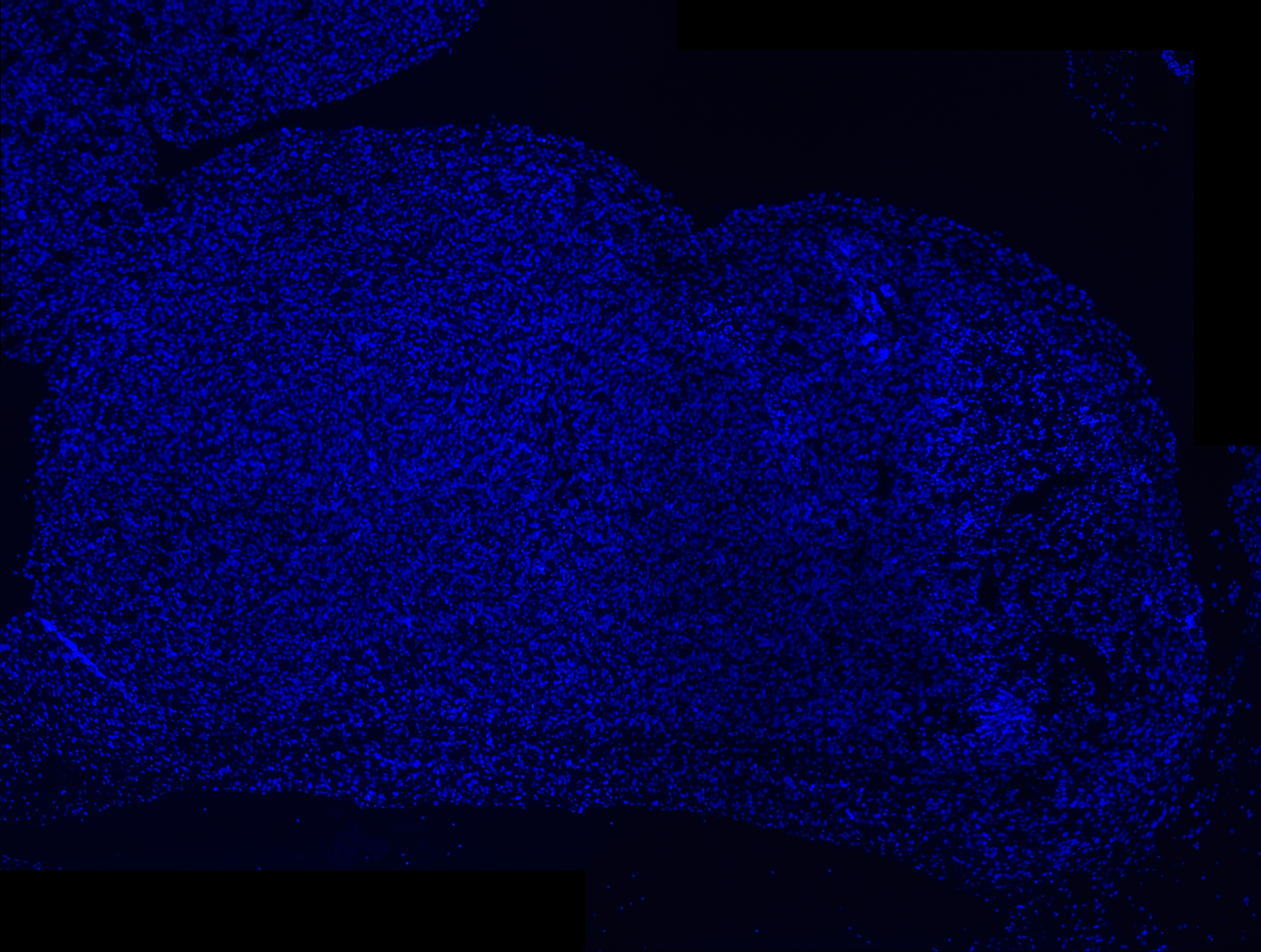

Supplement: Supplementary file 3 — Source Data for Appendix [file EMMM-12-e11416-s004.zip › Figure Appendix S6A/S6A_#6_LDM_CTX_+_MTD_CPB/Dapi.tif]

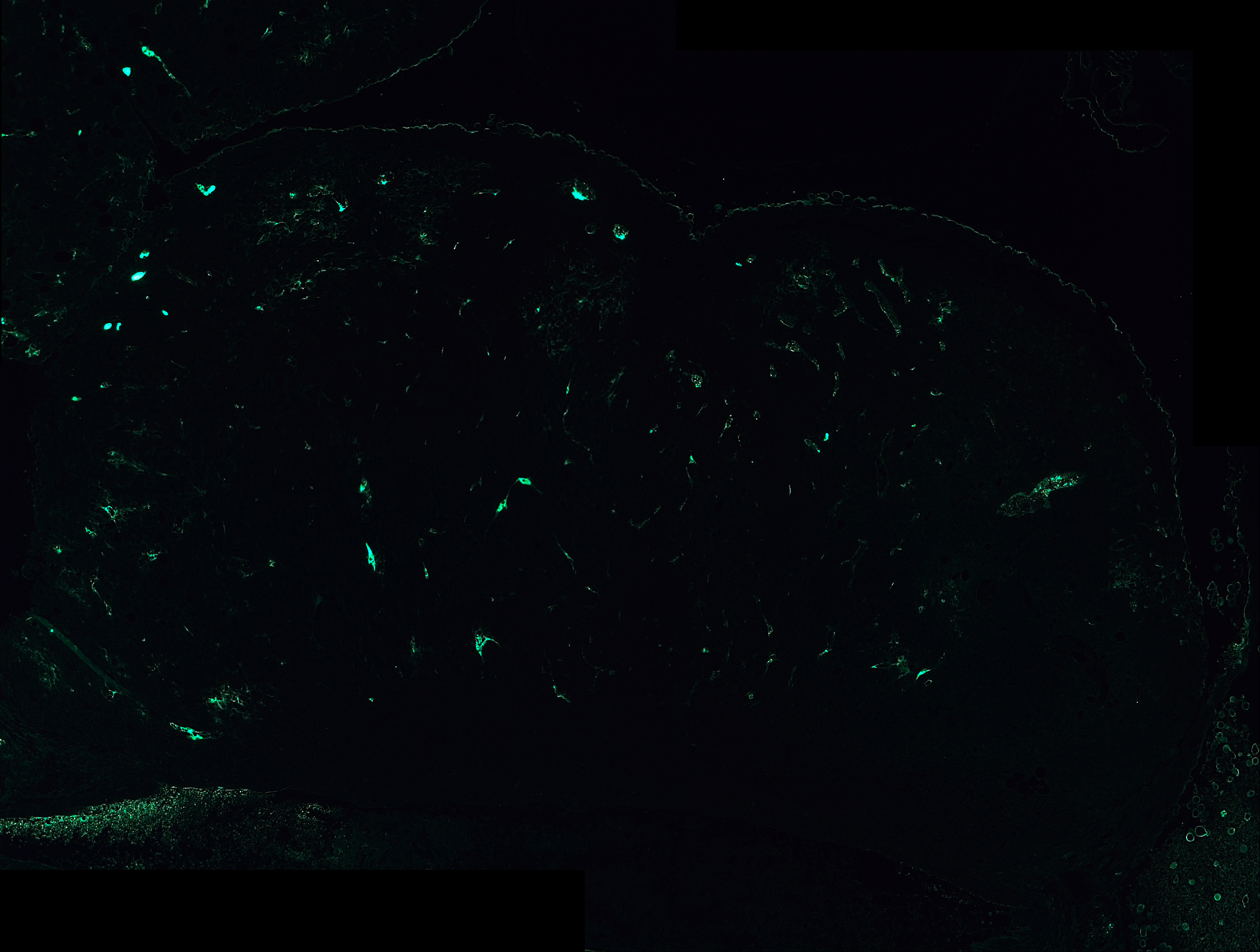

Supplement: Supplementary file 3 — Source Data for Appendix [file EMMM-12-e11416-s004.zip › Figure Appendix S6A/S6A_#6_LDM_CTX_+_MTD_CPB/Dextran.tif]

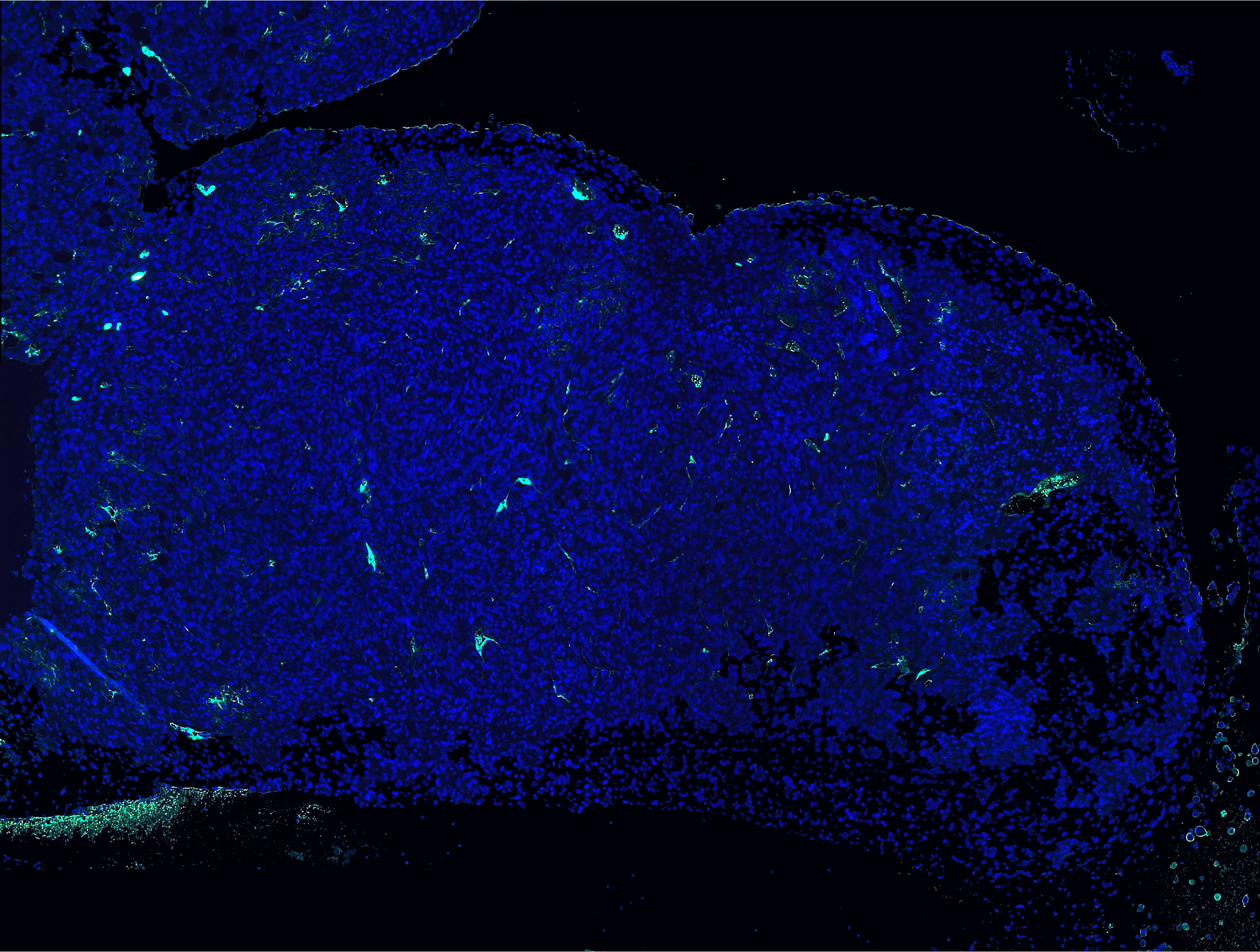

Supplement: Supplementary file 3 — Source Data for Appendix [file EMMM-12-e11416-s004.zip › Figure Appendix S6A/S6A_#6_LDM_CTX_+_MTD_CPB/Merged.tif]
